# Pax5 mediates the transcriptional activation of the *CD81* gene

**DOI:** 10.1038/s41598-021-02082-9

**Published:** 2021-11-25

**Authors:** Kohei Hosokawa, Hanako Ishimaru, Tadashi Watanabe, Masahiro Fujimuro

**Affiliations:** 1 Department of Cell Biology, Kyoto Pharmaceutical University, Misasagi-Shichonocho 1, Yamashinaku, Kyoto-shi, Kyoto, 607-8412 Japan; 2 Present Address: Department of Virology, Graduate School of Medicine, University of the Ryukyus, Okinawa, 903-0215 Japan

**Keywords:** Gene expression, Gene regulation, Transcriptional regulatory elements

## Abstract

CD81 is an integral membrane protein of the tetraspanin family and forms complexes with a variety of other cell surface membrane proteins. CD81 is involved in cell migration and B cell activation. However, the mechanism of the transcriptional regulation of the *CD81* gene remains unclear. Here, we revealed that CD81 transcriptional activation was required for binding of the transcription factor Pax5 at the Pax5-binding sequence (-54)GCGGGAC(-48) located upstream of the transcriptional start site (TSS) of the *CD81* gene. The reporter assay showed that the DNA sequence between − 130 and − 39 bp upstream of the TSS of the *CD81* gene had promoter activity for CD81 transcription. The DNA sequence between − 130 and − 39 bp upstream of TSS of *CD81* harbors two potential Pax5-binding sequences (-87)GCGTGAG(-81) and (-54)GCGGGAC(-48). Reporter, electrophoresis mobility shift, and chromatin immunoprecipitation (ChIP) assays disclosed that Pax5 bound to the (-54)GCGGGAC(-48) in the promoter region of the *CD81* gene in order to activate *CD81* transcription. Pax5 overexpression increased the expression level of CD81 protein, while the Pax5-knockdown by shRNA decreased CD81 expression. Moreover, we found that the expression level of CD81 was positively correlated with Pax5 expression in human tumor cell lines. Because CD81 was reported to be involved in cell migration, we evaluated the effects of Pax5 overexpression by wound healing and transwell assays. The data showed that overexpression of either Pax5 or CD81 promoted the epithelial cell migration. Thus, our findings provide insights into the transcriptional mechanism of the *CD81* gene through transcription factor Pax5.

## Introduction

CD81 from the tetraspanin family is composed of four transmembrane, three intracellular, and two extracellular domains. It is widely expressed in many tissues and is highly conserved in mammals^1^. One distinctive feature of tetraspanins is their ability to interact with other membrane proteins such as integrins, CD19, and other tetraspanin proteins^2^. Namely, tetraspanin forms multimolecular complexes with other tetraspanin proteins, which are known as tetraspanin-enriched microdomains in the cytoplasmic membrane. There have been few reports about the role of CD81 in B cell activation and cell migration; however, several studies about the molecular interaction of CD81 have been reported. CD81 transports CD19 to the cell surface from the endoplasmic reticulum via direct interaction with CD19 in B cells^3^. The co-receptor complex formed by CD81, CD19, and CD21 amplifies the B cell receptor (BCR) signaling. Mouse B cells derived from either CD19- or CD81-deficient mice were reported to display dysregulation of downstream signaling due to a defect in the organization of nano-clusters needed for an optimal response^4^. The engagement of CD81 on B cells induces ezrin tyrosine phosphorylation, leading to the facilitation of cytoskeletal reorganization^5,6^. CD81 is required for the formation of actin membrane protrusions and dendritic cell migration via Rac activation^7^. Moreover, CD81 increases melanoma cell motility by up-regulating metalloproteinase expression through Akt-dependent Sp1 activation signaling pathway^8^. Thus, CD81 regulates signal transduction and cell migration by interacting with other proteins^4,6–8^. CD81 expression on the oocyte surface was reported to be related to fertilization^9^. CD81 is also involved in virus infection and is related to HIV-1 and hepatitis C virus (HCV) infection^10,11^. CD81 was identified as a cellular receptor for hepatitis C virus^10^. It was reported that CD81 was involved in membrane fusion during the HIV-1 infection stage^11^ and incorporated into the viral membrane of HIV-1 during egress^12^.

As for the degradation of CD81, three studies have been reported. The gene related to anergy in lymphocytes (*GRAIL*) induces Lys48-linked poly-ubiquitination of CD81, causing CD81 instability^13^. Membrane-associated RING-CH (MARCH) induces the downregulation of CD81 from the cell surface and its relocalization to the lysosome^14^. The lysosome pathway degrades CD81 on the cell surface via clathrin-mediated endocytosis^15^. K63- and K29-linked poly-ubiquitination of Lys8 at the N-terminal intracellular domain of CD81 triggers the endocytosis of CD81^15^.

Thus, the functions and degradation machinery of CD81 have become clearer. In contrast to degradation, little is known about the transcriptional mechanism of CD81 expression. In particular, the transcriptional factor for CD81 expression and the promoter sequence of the *CD81* gene are unknown. We, therefore, attempted to address these unknowns and identified Pax5 as a key transcription factor for transcription of the *CD81* gene. Pax5 belongs to the paired box (*PAX*) family and functions as a transcriptional factor that plays an important role in B-cell differentiation^16^. Pax5 is required for upregulation of B-cell lineage-specific transcripts such as Iga (CD79a, mb-1)^17^, BLNK (SLP-65)^18^, and CD19^19^. These transcripts are involved in pre-BCR signaling and the developmental transition from pro-B cells to pre-B cells.

## Results

### Identification of the proximal promoter region of *CD81*

**Figure 1:**
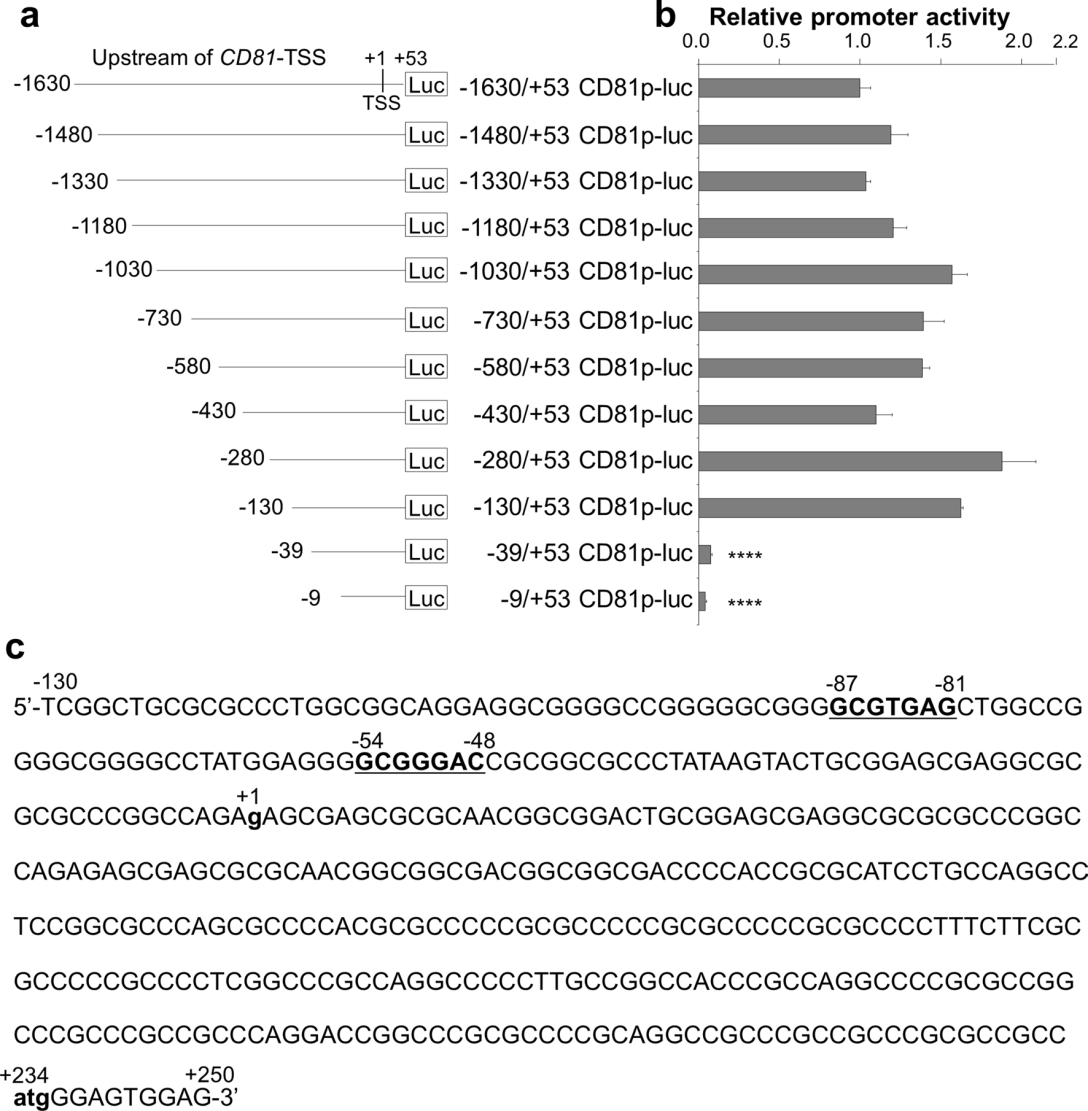
Identification of the proximal promoter region of the *CD81* gene. (**a**) Schematic diagrams of luciferase reporter plasmids to evaluate the transcriptional activity of the *CD81* gene 5’-flanking region. The DNA fragment between − 1630 bp upstream of *CD81*-TSS and + 53 bp downstream of *CD81*-TSS was cloned into the promoter-lacking firefly luciferase reporter plasmid (pGL4.11 [*luc2p*]), and the construct was designed as − 1630/ + 53CD81p-luc. Eleven 5’-deleted constructs (− 1480/ + 53, − 1330/ + 53, − 1180/ + 53, − 1030/ + 53, − 730/ + 53, − 580/ + 53, − 430/ + 53, − 280/ + 53, − 130/ + 53, − 39/ + 53, and − 9/ + 53CD81p-luc) were generated from − 1630/ + 53CD81p-luc. The transcriptional start site (TSS) of the *CD81* gene is defined as + 1. The luciferase gene is indicated as “*Luc*”. (**b**) Identification of the core promoter region in the *CD81* gene. ME180 cells were transiently transfected with the reporter and pSV-β-gal plasmids, and the luciferase activity was measured at 24 h after transfection. Transfection efficiency was normalized by the β-gal activity. The firefly luciferase activity/β-gal activity of − 1630/ + 53CD81p-luc was defined as 1.0. Values are shown as mean ± standard error of three independent experiments. (**c**) The DNA sequence of the 5’ flanking region (from + 250 to − 130 bp) of the *CD81* gene. The *CD81*-TSS, (+ 1)**g**, and the translational start codon, (+ 234)**atg**(+ 236), are shown as bold and lowercase. Two potential Pax5-binding sequences, (-87)**GCGTGAG**(-81) and (-54)**GCGGGAC**(-48), are formatted in bold and underline.

To identify the proximal promoter region required for *CD81* transcription, the upstream DNA sequence (1.63 kbp) of the *CD81* gene (NG_023386.1 Reference Sequence Gene) and its deleted sequences were used for the construction of luciferase reporter plasmids (Fig. 1a and Supplementary Fig. S1). The transcriptional start site (TSS) defined by reference to the database of TSS was indicated as “ + 1”. The start codon (+ 234) in the first exon was defined by reference to the GenBank human genomic clone (NM_004356.3). At first, we evaluated the promoter activity downstream (between + 1 and + 53 bp) of *CD81*-TSS. The DNA fragment between -1630 bp upstream of *CD81*-TSS and + 1, + 23, or + 53 bp downstream of *CD81*-TSS was cloned into the promoter-lacking reporter plasmid pGL4.11 [*luc2P*] (Supplementary Fig. S1a), and reporter plasmids (− 1630/ + 1, − 1630/ + 23, and − 1630/ + 53CD81p-luc) were transfected into ME180 and HeLa cells, followed by a reporter assay (Supplementary Fig. S1b). The region between − 1630 bp and + 53 bp (− 1630/ + 53 CD81p-luc) presented enough transcriptional activity in ME180 cells for the following experiments. Next, we evaluated which region of 1.63 kb upstream of *CD81*-TSS harbored the promoter activity. The *CD81*-TSS 5’-flanking region-deleted reporter plasmids (− 1480/ + 53, − 1330/ + 53, − 1180/ + 53, − 1030/ + 53, − 730/ + 53, − 580/ + 53, − 430/ + 53, − 280/ + 53, − 130/ + 53, − 39/ + 53, and − 9/ + 53CD81p-luc) were generated from − 1630/ + 53CD81p-luc (Fig. 1a) and transfected into ME180 cells. As a result, the region between − 1630 bp and − 130 bp upstream of *CD81*-TSS presented a 1- to 2-fold increase in transcriptional activity compared with -1630/ + 53 CD81p-luc. However, the region between − 39 and − 9 bp upstream of *CD81*-TSS presented a 10–20 fold decrease in transcriptional activity (Fig. 1b). These data indicate that the proximal promoter for *CD81* transcription is located between − 130 and − 39 bp upstream of TSS of the *CD81* gene. Therefore, we analyzed the potential transcription factor binding sites in this promoter region using the JASPER database. We found two potential Pax5-binding sites, (-87)GCGTGAG(-81) and (-54)GCGGGAC(-48), between − 130 bp and − 39 bp upstream of *CD81*-TSS (Fig. 1c).

### The proximal promoter of *CD81* contains a Pax5-binding site, and Pax5 is the transcription factor responsible for *CD81* promoter activation

**Figure 2:**
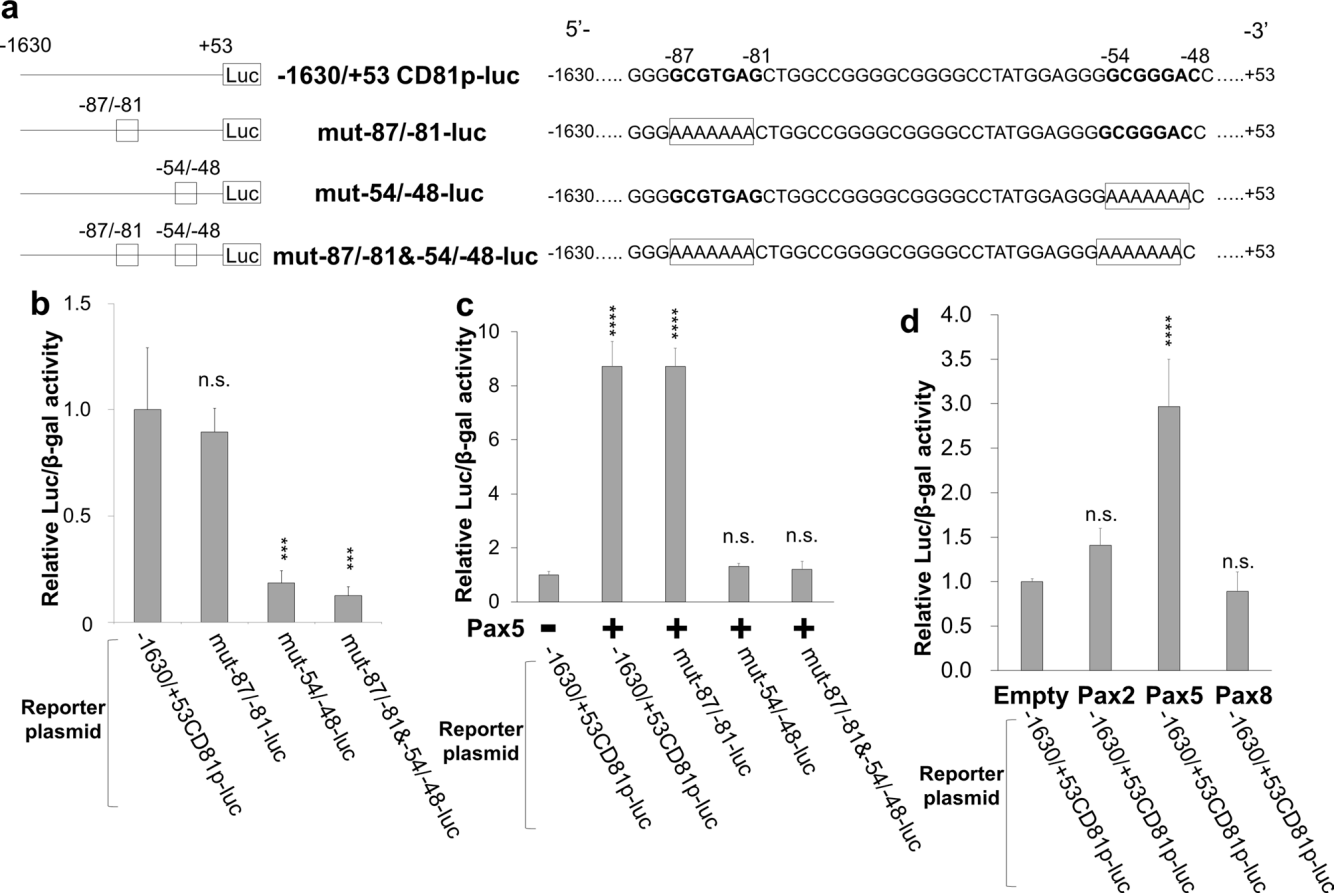
The Pax5 binding site, (-54)GCGGGAC(-48), in the *CD81* promoter is essential for transcriptional activation. (**a**) Diagrammatic representation of − 1630/ + 53CD81p-luc and mutated reporter plasmids that were generated from − 1630/ + 53CD81p-luc. The mut-87/-81-luc, mut-54/-48-luc, and mut-87/-81&54/-48-luc have AAAAAAA-substitutions in the Pax5-binding consensus sequences, (-87)GCGTGAG(-81), (-54)GCGGGAC(-48), and both of (-87)GCGTGAG(-81) and (-54)GCGGGAC(-48), respectively. The AAAAAAA-substituted Pax5 sites in the *CD81* promoter are indicated as open rectangles. (**b**) The Pax5-binding site, (-54)GCGGGAC(-48), is essential for *CD81* promoter activity. ME180 cells were transfected with the − 1630/ + 53CD81p-luc reporter plasmid or its mutant along with pSV-β-gal. After 24 h, cells were harvested, and luciferase and β-gal activities were measured. The luciferase/β-gal activity of − 1630/ + 53CD81p-luc was defined as 1.0. (**c**) The Pax5 is the transcription factor responsible for *CD81* promoter activation. HeLa cells were co-transfected with Pax5-pCIneo and − 1630/ + 53CD81p-luc (or its mutant) plasmids and cultured for 24 h. The luciferase/β-gal activity of − 1630/ + 53CD81p-luc without Pax5 was defined as 1.0. The mean ± SD are from three different experiments. (**d**) The activating effects of Pax5, Pax2 and Pax8 on *CD81* promoter in B cell. BC3 cells were co-transfected with − 1630/ + 53CD81p-luc and Pax expression plasmid and cultured for 24 h. The firefly luciferase/β-gal activity of − 1630/ + 53CD81p-luc without Pax was defined as 1.0.

Because the JASPER database suggested two potential Pax5-binding sites in the proximal promoter region (between − 130 and − 39 bp upstream of *CD81*-TSS), we asked whether mutation of either (or both) of the two Pax5-binding sites could affect its transcriptional activity. The AAAAAAA-substitutions in Pax5-binding sequences, (-87)GCGTGAG(-81) or/and (-54)GCGGGAC(-48), were generated from the − 1630/ + 53CD81p-luc plasmid (Fig. 2a). The mut-87/-81-luc, mut-54/-48-luc, and mut-87/-81&-54/-48-luc plasmids had AAAAAAA-substitutions in the Pax5-binding sites: (-87)GCGTGAG(-81), (-54)GCGGGAC(-48), and both of (-87)GCGTGAG(-81) and (-54)GCGGGAC(-48), respectively. The reporter assay revealed that mut-54/-48-luc and mut-87/-81&54/-48-luc displayed a 5- to 10-fold reduction in promoter activity compared with − 1630/ + 53CD81p-luc and mut-87/-81-luc (Fig. 2b). Furthermore, co-expression with Pax5 displayed an approximately ninefold increase in transcriptional activity compared with − 1630/ + 53CD81p-luc alone (Fig. 2c). Significantly, however, activation was not detected by co-expression with Pax5 and either mut-54/-48-luc or mut-87/-81&54/-48-luc. These data indicate that a Pax5 core binding sequence, (-54)GCGGGAC(-48), is necessary for exerting the promoter activity of the − 1630/ + 53CD81p-luc plasmid. In other words, the proximal promoter of the *CD81* gene (the region from + 53 to − 130 bp of *CD81*-TSS) contains a Pax5-binding site, (-54)GCGGGAC(-48), for *CD81* transcription. Pax5 is therefore one of the master transcription factors of *CD81* gene expression.

The mammalian *PAX* genes are classified into four sub-groups (i.e., sub-groups 1, 2, 3, and 4). Pax5 belongs to sub-group 2, to which Pax2 and Pax8 also belong. Therefore, we evaluated the participation of Pax2 and Pax8 in *CD81* promoter activation by conducting a reporter assay using B-lymphoma cells. Reporter plasmid (− 1630/ + 53CD81p-luc) and Pax (or empty) plasmid were co-transfected into BC3 cells. Pax2 expression increased transcriptional activity by about 40% compared with empty vector, whereas Pax8 had no effect on transcriptional activity (Fig. 2d). In contrast to Pax8, Pax5 increased transcriptional activity by about 3 times compared with empty vector. These suggest that Pax5 mainly induces to activate the CD81 transcription, and Pax2 also might be involved in the CD81 transcriptional activation.

### Pax5 binds to (-54)GCGGGAC(-48) within the proximal promoter of *CD81*

**Figure 3:**
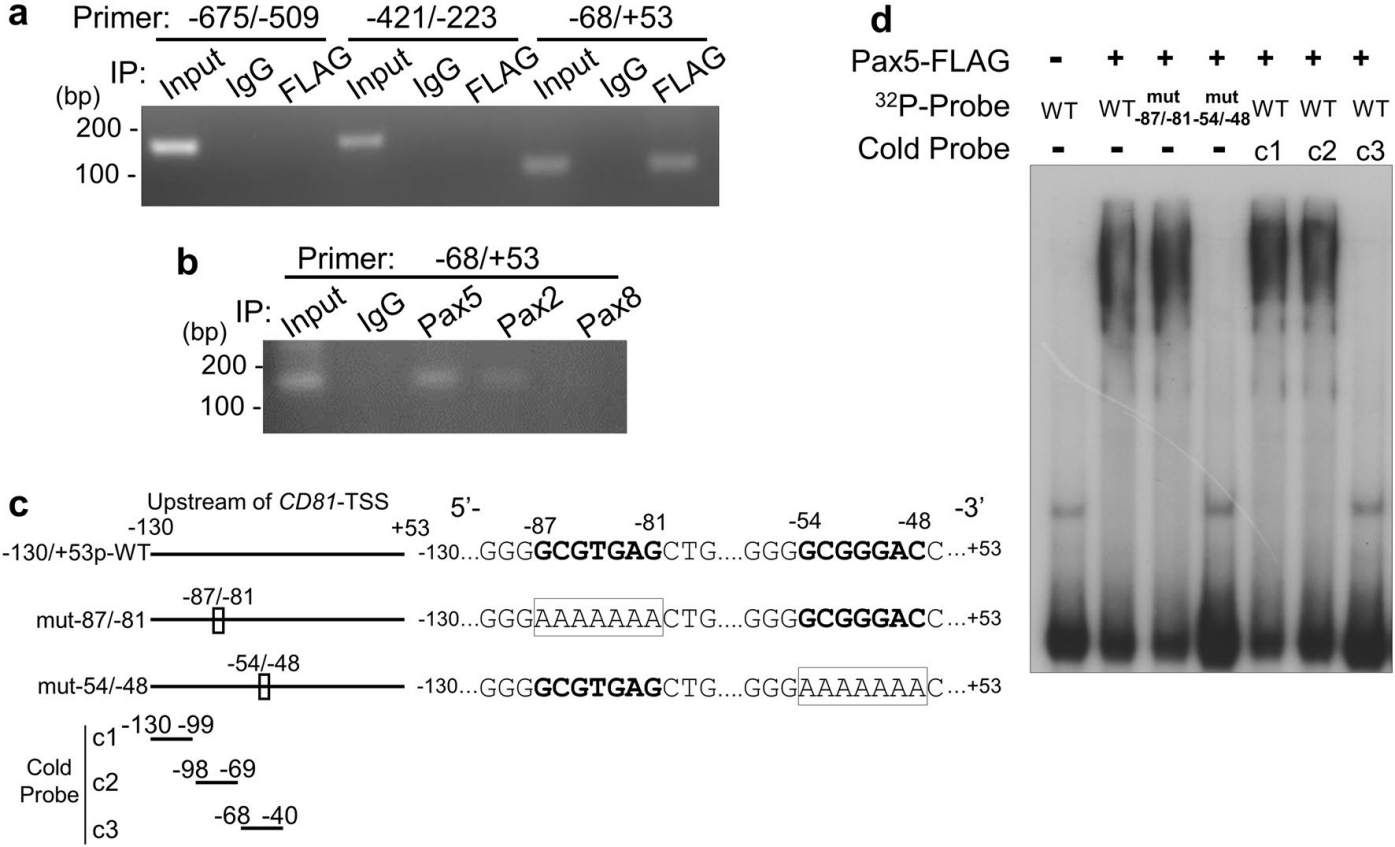
Pax5 binds to (-54)GCGGGAC(-48) but not (-87)GCGTGAG(-81) within the *CD81* promoter. (**a**) ChIP analysis of Pax5-binding to the upstream region of *CD81*-TSS. HeLa cells were transfected along with the Pax5-FLAG expressing plasmid, and after 24 h, cell extracts were prepared. Pax5-FLAG bound DNA was immunoprecipitated with FLAG-beads, and co-precipitated DNA was analyzed by PCR using primers targeting the upstream region of *CD81*-TSS: − 675/ − 509, − 421/ − 223, and − 68/ + 53. Original image of ChIP assay is shown in Supplementary Fig. S2a. (**b**) Endogenous Pax5 binds to the region from − 68 to + 53 bp in B cells. Akata cells were subjected to a ChIP assay using anti-Pax2, -Pax5, or -Pax8 antibodies. Immunoprecipitated Pax2, Pax5, and Pax8 samples from cell extracts of Akata B cells were analyzed by PCR using primers targeting the upstream region of *CD81*-TSS between − 68 and + 53 bp (− 68/ + 53). An original image of the ChIP assay is shown in Supplementary Fig. S2b. (**c**) Schematic representation of ^32^P-labelled and cold DNA probes for EMSA to analysis Pax5-binding to two potential Pax5-binding sites. The wild-type (WT) probe, − 130/ + 53p-WT, consisted of the *CD81* proximal promoter containing two potential Pax5-binding sequences. The mutated probes, mut-87/-81 and mut-54/-48, contained the AAAAAAA-substitutions in the consensus Pax5-binding sequence, (-87)GCGTGAG(-81) and (-54)GCGGGAC(-48), respectively. (**d**) EMSA of Pax5-binding to the two Pax5-binding consensus sequences. 293T cells were transfected along with Pax5-FLAG plasmid, and FLAG-tagged Pax5 protein was purified by anti-FLAG antibody-immobilized beads from transfected cell extracts. The purified Pax5-FLAG and ^32^P-labelled DNA probe were incubated at room temperature for 20 min to form a DNA–protein complex. In competition experiments, a 100-fold molar excess of unlabeled cold oligonucleotide duplexes (c1, c2, or c3) was added during the preincubation period. The incubated samples were subjected to native PAGE. Original image of EMSA is shown in Supplementary Fig. S2c.

We also attempted to disclose which potential Pax5-binding sites, (-87)GCGTGAG(-81) or (-54)GCGGGAC(-48), was the target of Pax5 by ChIP assay and EMSA (Fig. 3). HeLa cells were transfected with Pax5-3xFLAG plasmid, and cell lysate was subjected to ChIP assay using anti-FLAG antibody immobilized beads for precipitation of Pax5-conjugated DNAs. The precipitated Pax5-conjugated DNAs were amplified by PCR using a primer set for detecting the upstream region of *CD81*-TSS between − 675 and − 509 bp (− 675/− 509), − 421 and − 223 bp (− 421/− 223), or -68 and + 53 bp (− 68/ + 53). As a result, the binding of Pax5 to − 68/ + 53 DNA region was detected by PCR, while the binding of Pax5 to neither − 675/− 509 DNA region nor − 675/− 509 DNA region was detected (Fig. 3a). Data means that Pax5 binds to the *CD81*-TSS-upstream region from − 68 to + 53 bp. The upstream region of *CD81*-TSS from − 68 to + 53 bp contains (-54)GCGGGAC(-48) but not (-87)GCGTGAG(-81), meaning that the (-54)GCGGGAC(-48) sequence in CD81 promotor is one of the Pax5-binding sites. Moreover, we tested whether endogenous Pax2, Pax5, and Pax8 bound to the region from − 68 to + 53 bp, which contains the Pax5 core binding site (-54)GCGGGAC(-48) in B-lymphoma cells under physiological conditions (Fig. 3b). The cell extract from Akata cells was subjected to a ChIP assay using anti-Pax2, Pax5, or Pax8 antibody-immobilized beads. Immunoprecipitated endogenous Pax-conjugated DNAs were amplified by PCR using a primer set for detecting the upstream region of *CD81*-TSS between − 68 and + 53 bp (− 68/ + 53). The data showed that endogenous Pax5 bound to the region from − 68 to + 53 bp in B cells under normal conditions, while Pax2 also weakly bound to this region. Taken together with data in Fig. 2d, these indicate that Pax5 induces CD81 transcriptional activation, and Pax2 also partly contributes to it.

To reveal which potential Pax5-binding sites, (-87)GCGTGAG(-81) or (-54)GCGGGAC(-48), is the true target of Pax5, we analyzed the direct binding of Pax5 to these DNA sequences by EMSA. The DNA probe (− 130/ + 53p-WT) was prepared by digestion of − 130/ + 53CD81p-luc plasmid and labeling with ^32^P (Fig. 3c). The wild-type (WT) probe, − 130/ + 53p-WT, contained two potential Pax5-binding sites in the proximal promoter of the *CD81* gene. The mutated probes, mut-87/-81 and mut-54/-48, contained the AAAAAAA-substitutions in two potential Pax5-binding sites, (-87)GCGTGAG(-81) and (-54)GCGGGAC(-48), respectively. When purified Pax5 protein and each ^32^P-labeled probe were incubated, the gel shift of either − 130/ + 53p-WT or mut-87/-81 DNA probe by Pax5-complex formation was detected; however, the gel shift of mut-54/-48 DNA probe was not detected (Fig. 3d). Moreover, competition with unlabeled cold probes of c1 (from − 130 to − 99 bp) and c2 (from − 98 to − 69 bp) did not abolish the complex formation, whereas competition with cold probes c3 (from − 68 to − 40 bp) completely inhibited the complex formation. The results of reporter assay (Fig. 2), ChIP, and EMSA suggest that the *CD81* proximal promoter (between − 130 and − 39 bp upstream of *CD81*-TSS) harbors the Pax5 core binding sequence (-54)GCGGGAC(-48), and Pax5 protein binds to (-54)GCGGGAC(-48) in the *CD81* promoter, leading to *CD81* transcription.

### The transcription factor Pax5 increase the CD81 expression

**Figure 4:**
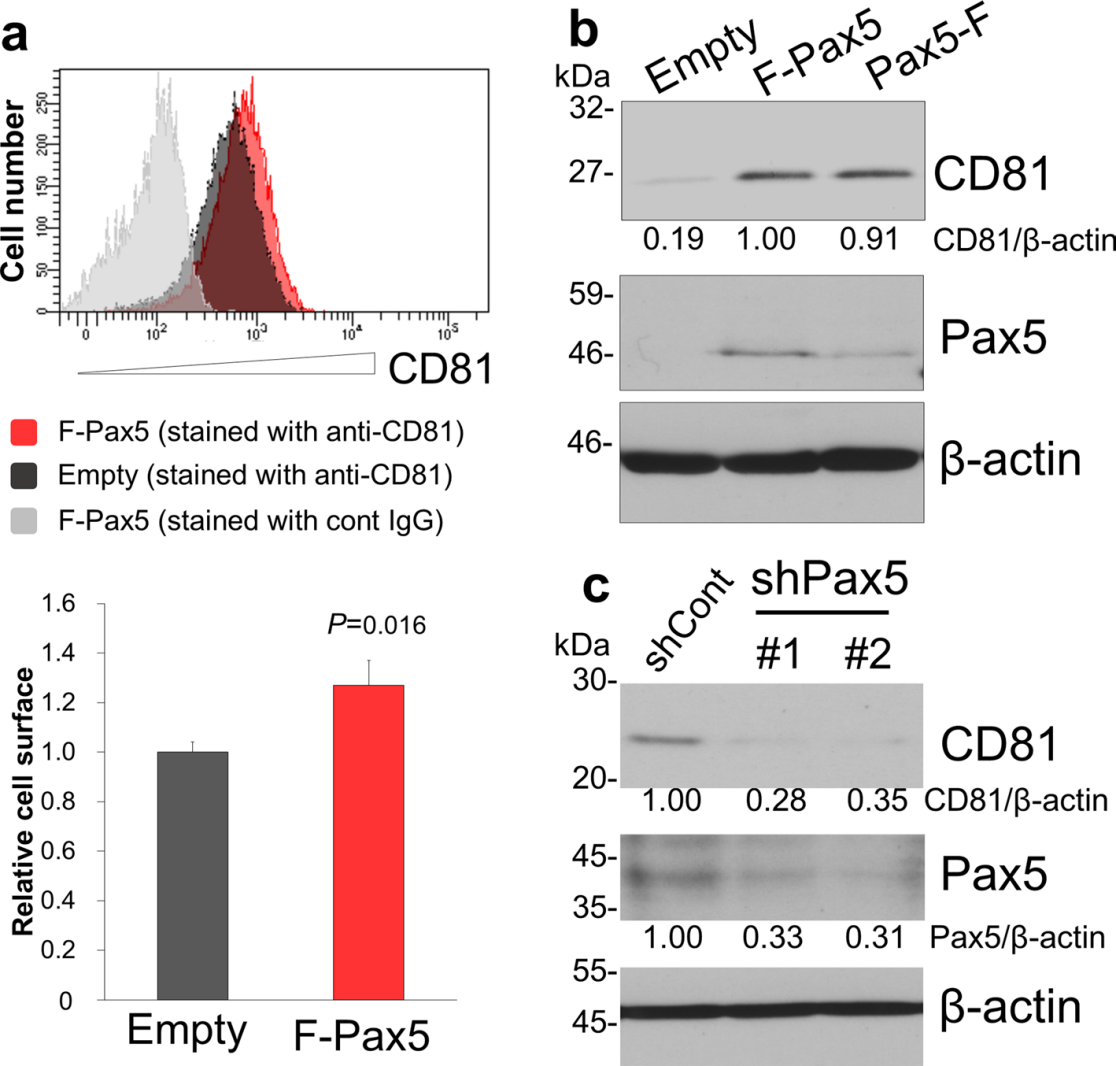
Pax5 up-regulates the CD81 expression. (**a**) The effect of exogenous Pax5 expression on plasma membrane-bound CD81 expression in HeLa cells, in which CD81 was poorly expressed. HeLa cells were transfected with the FLAG-Pax5 expression plasmid and cultured for 24 h. CD81 expression on the cell surface was measured by flow cytometry. The gray histogram indicates the isotype control. Red and black histograms show CD81 expressing cells in Pax5-transfected cells and control cells, respectively. The mean fluorescence intensities of control cells and Pax5 transfected cells are shown in the black and red bar graphs, respectively (bottom panel). (**b**) Effect of exogenous Pax5 expression on CD81 expression in whole HeLa cells. Cells were transfected with FLAG-Pax5 or Pax5-FLAG and cultured for 24 h. Cell lysates were subjected to Western blotting with anti-CD81 antibodies. For preparation of SDS-PAGE sample, the sample buffer without 2-mercaptoethanol was used, because anti-CD81 antibody (Santa Cruz Biotechnology) recognizes non-reduced form of CD81. (**c**) Effect of Pax5 knockdown by shRNA on CD81 expression in ME180 cells, in which CD81 was highly expressed. Cells were transfected with shPax5 targeting Pax5 or shCont targeting GFP and cultured for 48 h. CD81 in whole ME180 cells was detected by immunoblotting. (**b** and **c**) Original images of Western blotting are shown in Supplementary Fig. S3.

Because the activation of *CD81* transcription was required for Pax5 binding in the *CD81* promoter, the effect of expression (or elimination) of Pax5 on CD81 expression in low CD81-expressing cells was analyzed. We evaluated whether exogenous Pax5 overexpression could upregulate CD81 expression in HeLa cells, in which CD81 was poorly expressed. CD81 expression on the cell surface and in whole HeLa cells was quantified by flow cytometry and Western blotting, respectively. Overexpression of FLAG-tagged Pax5 increased CD81 protein expression on the cell surface (Fig. 4a) as well as in whole HeLa cells (Fig. 4b). We also examined the effect of transient Pax5-knockdown using shRNA on CD81 expression in ME180 cells. The Pax5-knockdown by shRNA (shPax5) decreased CD81 expression along with Pax5 downregulation in ME180 cells (Fig. 4c). These results strongly support that CD81 expression is enhanced by Pax5.

**Figure 5:**
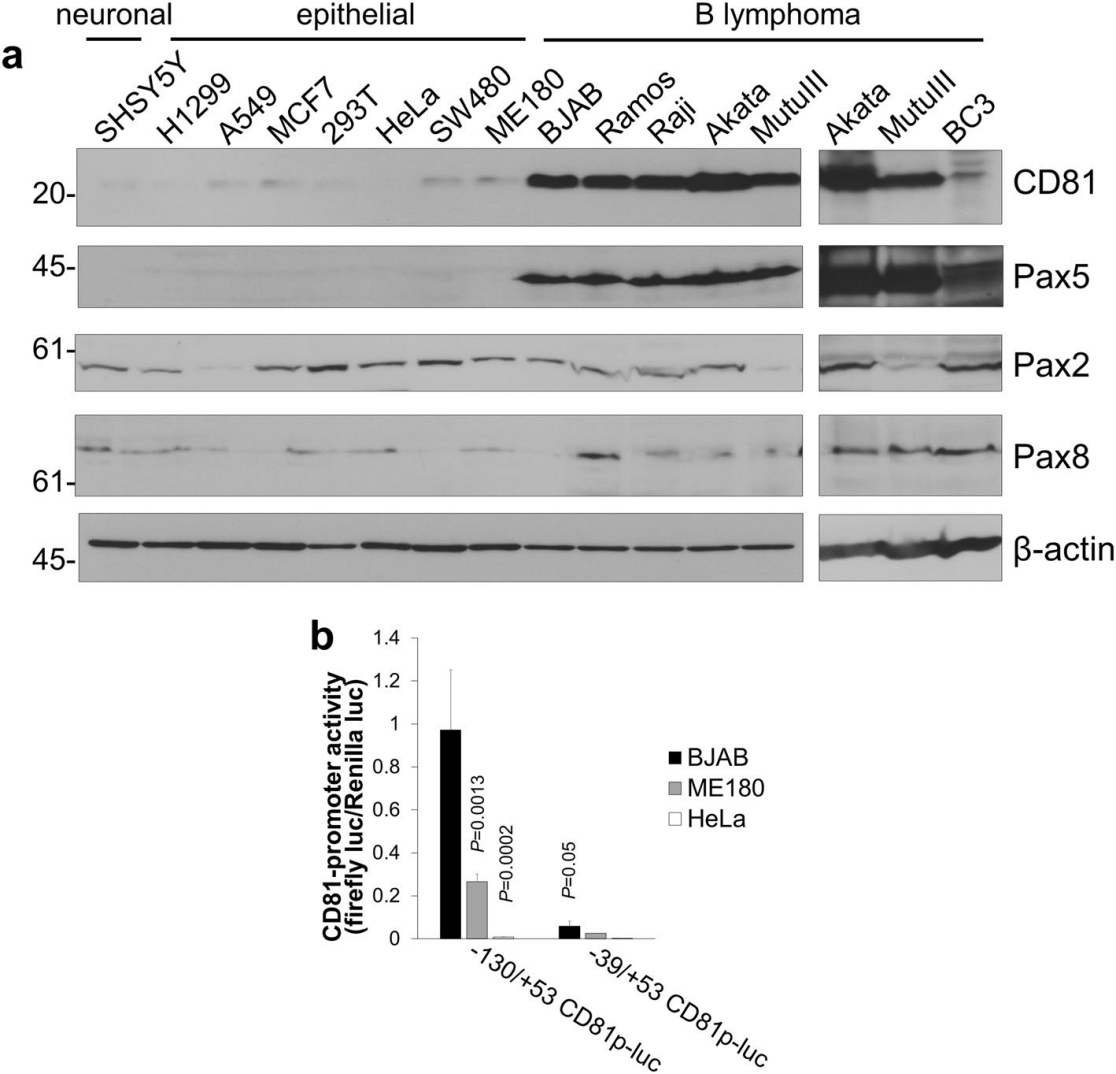
Expression levels of CD81 and Pax5 in human tumor cells. (**a**) Expression of CD81 and Pax5 proteins in human neuronal (SHSY5Y), epithelial (H1299, A549, MCF7, 293T, HeLa, SW480, and ME180), and B-lymphoma (BJAB, Ramos, Raji, Akata, MutuIII and BC3) cells. Each cell extract was subjected to Western blotting using anti-CD81, anti-Pax5, anti-Pax2 and anti-Pax8 antibodies. When we detected CD81 by anti-CD81 antibody, the sample buffer without 2-mercaptoethanol for the sample preparation was used. Original images of Western blotting are shown in Supplementary Fig. S4. The long exposure images of CD81 and Pax5 blotting using ME180, H1299, and HeLa cells are shown in Supplemental Fig. S5. (**b**) The transcriptional activity of the *CD81* promoter in BJAB, ME180, and HeLa cells. Cells were transfected with *CD81* proximal promoter-containing − 130/ + 53CD81p-luc plasmid and pRL-CMV-Luc, harvested at 24 h after transfection, and subjected to a dual-luciferase assay. The pRL-CMV-Luc plasmid was used as an internal control and contained the CMV IE-promoter and the *Renilla* luciferase gene. Firefly luciferase activity was normalized to *Renilla* luciferase activity. The *CD81* promoter-lacking − 39/ + 53 CD81p-luc plasmid was used as a negative control.

To obtain further evidence that Pax5 induces the CD81 expression, we evaluated the correlation between CD81 and Pax5 in human tumor cell lines derived from different tissues: neuronal cell (SHSY5Y), epithelial cells (H1299, A549, MCF7, 293T, HeLa, SW480, and ME180), and B-lymphoma cells (BJAB, Ramos, Raji, Akata, MutuIII and BC3). High expression of both CD81 and Pax5 was detected in B-lymphoma cell lines (Fig. 5a), whereas neither CD81 nor Pax5 was detected in neuronal cells or epithelial cells (H1299, 293T, and HeLa). The long exposure images of CD81 and Pax5 blotting using ME180, H1299, and HeLa cells are shown in Supplemental Fig. S5. The low expression of CD81 and Pax5 was detected in ME180 cells. In addition to Pax5, expression of other sub-group 2 members (Pax2 and Pax8) was also analyzed. Pax2 was moderately expressed in the tested cell lines, except for A549 cells derived from lung cancer tissue. Pax8 exhibited a low level of expression in all tested cell lines. We next sought to clarify whether these differences in CD81 expression were due to endogenous Pax5 expression levels in each cell line. To this end, we validated the *CD81* transcriptional activity in an epithelial cell line (ME180 and HeLa) and a B-cell line (BJAB) by carrying out a reporter assay using the − 130/ + 53CD81p-luc reporter plasmid, which contains the proximal *CD81* promoter and a Pax5-binding site, (-54)GCGGGAC(-48) (Fig. 5b). The − 39/ + 53 CD81p-luc plasmid, lacking a Pax5-binding site, was used as a negative control. As expected, BJAB cells transfected with − 130/ + 53p-luc plasmid showed an increase in transcriptional activity compared with HeLa and ME180 cells, which lends support to the notion that there is a correlation between CD81 and Pax5 expression. The results shown in Figs. 3, 4, and 5 strongly support the hypothesis that Pax5 is one of the master transcription factors responsible for the activation of CD81 transcription.

### Exogenous expression of Pax5 promotes cell migration and membrane protrusion formation

**Figure 6:**
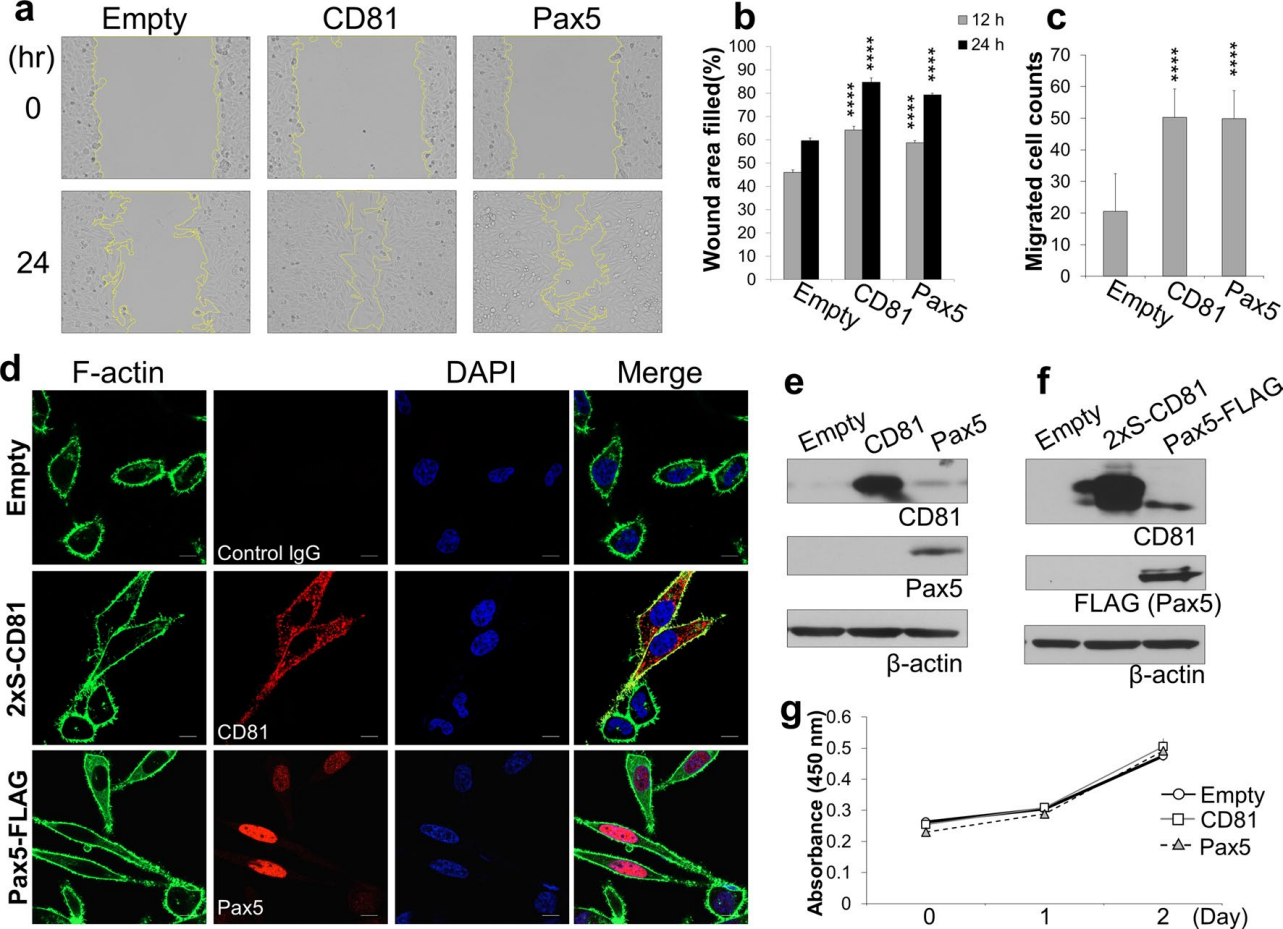
Pax5 expression enhances CD81-dependent functions: cell migration and membrane protrusion formation. (**a**, **b**) The effect of Pax5 expression on wound healing of HeLa cells on a cells sheet. HeLa cells were transfected with empty, CD81, or Pax5 plasmids and cultured for 12 h. The confluent cell sheet was scratched with a P2 micro-pipette tip, and this timing was defined as 0 h. The wound healing area was enclosed with a yellow line and measured using ImageJ. Left pictures are representative images of the wound healing assay at 0 and 24 h. (**b**) The graph shows the quantitative data of the wound healing filled area. The area was defined as 100% when all the scratches are filled, i.e., the wound area at 0 h was defined as 100% in each cell group. Wound area filled (%) = (mean of first wound area—mean of last wound area)/first wound area × 100. The gray and black bars show the wound area filled at 12 and 24 h, respectively. (**c**) Evaluation of cell migration activity in Pax5-transfected cells by a transwell migration assay without matrigel. HeLa cells were transfected with empty, CD81, or Pax5 plasmids and cultured for 24 h. Transfected cells were seeded in the top chamber of the transwell insert. The top and bottom chambers were filled with culture medium in 10% FBS. Cells were allowed to migrate for 48 h. The migrated cells in the lower chamber were counted. (**d**) Microscopic visualization of actin-based membrane protrusions. HeLa cells were transfected with empty vector, 2xS-CD81, or Pax5-FLAG. Fixed and permeabilized cells were stained with Alexa488-conjugated phalloidin for detection of F-actin (green) and either mouse anti-S-probe (CD81) or mouse anti-FLAG (Pax5) monoclonal antibodies. Stained cells were incubated with Alexa594-conjugated anti-mouse Ig antibodies (red), and nuclei were stained with DAPI. (**e** and **f**) Validation of the Pax5-induced increase of CD81 expression. Figure 6e and f show Western blotting of Pax5 and CD81 using the HeLa cells prepared in Fig. 6c and d, respectively. Original images of Western blotting are shown in Supplementary Fig. S6. (**g**) The effects of Pax5 and CD81 expression on the proliferation of HeLa cells.

We found that Pax5 expression increased the CD81 expression. CD81 is reported to promote the formation of actin-based membrane protrusion, cell migration, and metastasis^7,20^. Because Pax5 expression is expected to activate CD81 function as well as CD81 expression, we investigated the effects of exogenous Pax5 expression on CD81-dependent function (*i.e.*, on cell migration and formation of membrane protrusion). CD81-nonexpressing HeLa cells were transfected with Pax5 or CD81 and analyzed for cell migration ability by the wound healing assay. Either Pax5 or CD81 expression exhibited a significant increase in migration ability compared with mock transfected cells (Fig. 6a and b). The transwell migration assay also revealed that the migration ability of both Pax5 and CD81 transfected HeLa cells was much higher than that of mock transfected cells (Fig. 6c). To gain insight into the molecular mechanisms through which CD81 and Pax5 enhances the migration ability, we monitored the change in cell morphology and filamentous actin (F-actin) distribution by CD81 and Pax5 expression. HeLa cells were transfected with 2xS-CD81 or Pax5-FLAG, and F-actin distribution in the transfected cells was observed by IFA. The Pax5 expression as well as CD81 expression increased the cell spreading along with the development of motile subcellular structures such as membrane protrusions (Fig. 6d). CD81 or Pax5 expression, therefore, visibly rendered HeLa cells more motile. In addition, we confirmed that Pax5 expression increased the CD81 expression by Western blotting using a part of HeLa cells prepared in Fig. 6c and d. Figure 6e and f show blotting data using the cell lysate prepared in Fig. 6c and d, respectively. An increase in CD81 expression was detected by exogenous Pax5 expression compared with empty plasmid (the right lanes of Fig. 6e and f). These results indicated that Pax5 induced CD81 expression, resulting in increased cell motility and spreading. Moreover, a small amount of CD81 may be enough for activating cell migration. We also confirmed that Pax5 and CD81 expression did not affect the proliferation of HeLa cells (Fig. 6g).

## Discussion

**Figure 7:**
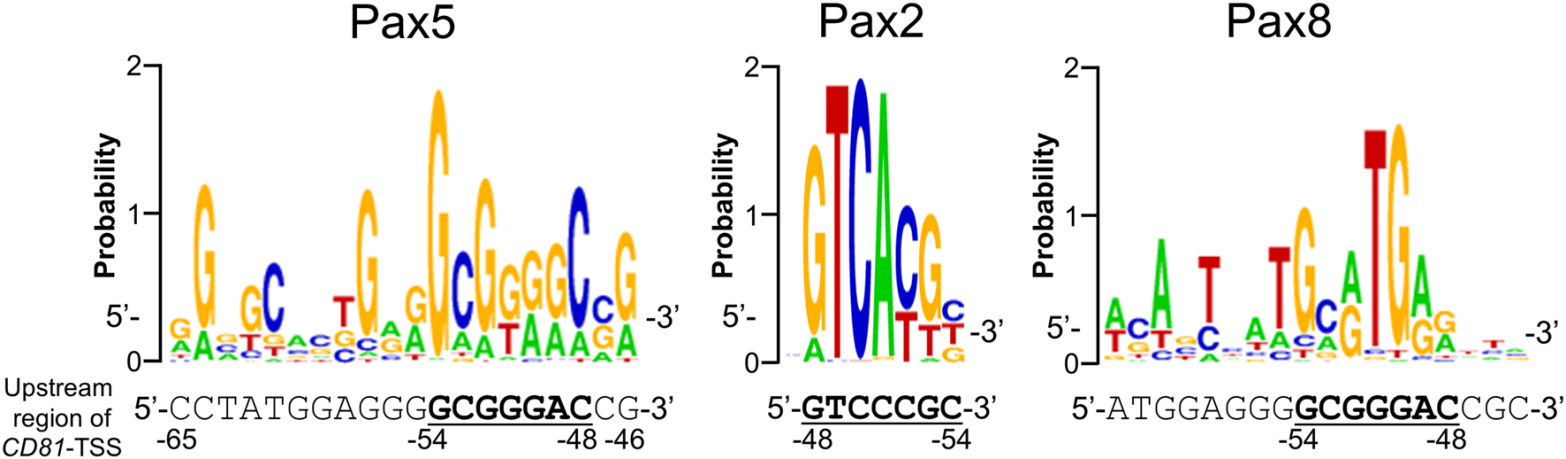
Pax5 consensus binding sequences and Pax5 core binding site in the promoter region of the *CD81* gene. Pax5, Pax2, and Pax8 consensus binding sequences are shown as sequence logos, which were generated using ISMARA (Integrated System for Motif Activity Response Analysis) (https://ismara.unibas.ch/mara/). For each position, the percentage occurrence of each DNA base is indicated. The lower DNA sequence of the Pax5 logo is the Pax5 consensus binding site, (-65)CCTATGGAGGG**GCGGGAC**CG(-46), within the promoter region of the *CD81* gene. The Pax5 core binding sequence, (-54)**GCGGGAC**(-48), is shown in bold text and underlined. The lower DNA sequence of the Pax2 logo is the complementary sequence of (-54)GCGGGAC(-48). The (-54)GCGGGAC(-48) 7 bp sequence, which we identified as the Pax5 core binding site, matched 100% (7/7), 85.7% (6/7), and 71.4% (5/7) of the consensus binding sequence of Pax5, Pax2, and Pax8, respectively.

In mammals, there are nine paired box (*PAX*) genes. All Pax transcription factors have a paired DNA binding domain formed by two helix-turn-helix motifs^16^. These nine PAX genes are classified into four sub-groups (Pax groups 1, 2, 3, and 4) according to the presence or absence of other domains, such as the octapeptide motif and the homeodomain. The octapeptide motif is involved in transcriptional repression activity and the recruitment of Groucho/Grg4 co-repressors^16,21^, while the homeodomain is involved in the enhancement of DNA-binding activity^16,22^. Pax5, Pax2, and Pax8 belong to the Pax group 2, which contains a paired DNA binding domain, an octapeptide motif, and a partial homeodomain. Pax2 is related to chromosomal translocation in tumor cells and the development of kidney, central nervous system (CNS), and optic nerve, while Pax8 is involved in thyroid, CNS, and kidney cell development^23,24^. In this study, we revealed that the proximal promoter for CD81 transcription is located between − 130 and − 39 bp upstream of TSS of the *CD81* gene. Moreover, the (-54)GCGGGAC(-48) sequences in the proximal *CD81* promoter are essential for the binding of Pax5 and CD81-transcription. To the best of our knowledge, this is the first report to demonstrate a master transcription factor and its binding sequence in the promoter for *CD81* gene expression. The Pax5 consensus binding sequence is shown as a sequence logo (Fig. 7), which was identified by the Integrated System for Motif Activity Response Analysis (ISMARA). The Pax5 consensus binding sequence identified by ISMARA is in agreement with the consensus binding sequence proposed by an earlier report based on experimental data^25^. The sequence (-65)CCTATGGAGGG**GCGGGAC**CG(-46) within the promoter region in the *CD81* gene is shown at the bottom of the sequence logo. Pax2 and Pax8 consensus binding sequence logos are also presented for reference. The (-54)GCGGGAC(-48) 7 bp sequence, which we identified as the Pax5 core binding site, matched 100%, 85%, and 71% of the consensus binding sequences of Pax5, Pax2, and Pax8, respectively. These matching-percentages lend support to our data showing their binding ability with the *CD81* proximal promoter (Fig. 3b).

In addition to Pax5, the JASPER database suggested that the upstream DNA sequence (1.63 kbp) of the *CD81* gene contains one YY1- (from − 822 to − 815 bp), two GATA3- (from − 1224 to − 1219 bp and − 368 to − 363 bp), two ZEB1- (from − 962 to − 954 bp and − 199 to − 189 bp), and two TCF3-binding consensus sequences (from − 1474 to − 1465 bp and − 199 to − 190 bp). The deletion of these transcription factor-binding sequences did not significantly affect the luciferase gene expression (Fig. 1b). These data provide support to that Pax5 is a transcription factors responsible for *CD81* promoter activation. However, we identified the *CD81* proximal promoter and Pax5 as the responsible transcription factor by reporter assay and EMSA using cells cultured under normal conditions (*i.e.*, not special condition). Therefore, we need to consider the regulation of *CD81* transcription by transcription factors other than Pax5 under special conditions, such as cytokine stimuli, stress responses, viral infection, and B-cell developmental stages.

High levels of *CD81* gene expression can be detected during B-cell development^26^ (*i.e.*, in hematopoietic stem cell (HSC), pre-pro-B, pro-B, pre-B, early-B, and immature-B cell stages). CD81 expression is further upregulated at specific stages: the transition from pre-pro-B to pro-B cells and the transition from pre-B to early-B cells^26^. In addition, it has been reported that CD81 expression is epigenetically regulated by DNA methylation of the CpG island region of the *CD81* gene^27^. Paiva et al. found that CD81 protein expression levels are inversely correlated with DNA methylation levels in the CpG island region of the *CD81* gene^27^. They also reported the location of the CpG island of the *CD81* gene. Cells with high expression levels of CD81 exhibit low levels of methylation of the *CD81* gene; therefore, it is thought that Pax5 preferentially binds to the CpG island of the *CD81* gene and induces CD81 expression. Interestingly, the CpG islands of the *CD81* gene identified by Paiva and colleagues contained the (-54)GCGGGAC(-48) sequence, which we identified as the Pax5-binding site. Therefore, there seems to be reasonable agreement between the Pax5-binding site and the epigenetically controlled *CD81* promoter region.

Regarding B-cell developmental stages, Revilla-i-Domingo et al. and Schebesta et al. identified the Pax5-dependent activated genes between pro-B and mature-B cell stages using Pax5 + / + and Pax5 − / − cells^28,29^. However, the Pax5-dependent activated genes that they reported did not include the *CD81* gene. For CD81 transcriptional activation during the transition from pro-B to mature-B cells, we must consider the contribution of other transcription factors including YY-1, GATA3, and Pax2, which are mentioned above. Moreover, epigenetic regulation by DNA methylation of the CpG island in the CD81 promotor may be involved in CD81 expression during B cell development. During HSC, pro-B, pre-B, early-B, and immature-B cell stages, CD81 expression is already upregulated^26^. This may also be one of reasons why CD81 was not identified as a Pax5 differentially expressed gene at the transition from pro-B to mature-B cells.

Pax5 functions as a transcription factor that is important for commitment to the B-lymphoid lineage^30–32^. Pax5 also functions as either a transcriptional activator or repressor^17–19,21,32^. The *CD19* gene is one of the genes that Pax5 upregulates^19^. CD19 and CD81 collaboratively regulate B-cell receptor (BCR) signaling. CD81 interacts with CD19 and is involved in BCR signaling amplification upon surface-bound antigen stimulation^4^. Our study provided evidence that the *CD81* gene is also one of the genes that Pax5 upregulates. Moreover, Pax5 is highly expressed in many B-cell lines. Thus, it seems reasonable to conclude that Pax5 upregulates the transcription of CD81, which is a key component of the BCR signal molecule.

CD81 membrane protein promotes melanoma cell motility by inducing metalloprotease MT1-MMP expression through the Akt-dependent Sp1 activation signaling pathway^8^. Moreover, CD81 contributes to the migration of dendritic cells and the motility of breast cancer cells^7,33^. In dendritic cells, CD81 is important for the formation of Rac-dependent actin membrane protrusions^7^. Fibroblast-secreted CD81-positive exosomes enhance the metastatic potential of breast cancer cells by regulating their motility^33^. In addition, Pax5 was reported to activate migration gene in B lymphocytes^29^. We found that overexpression of not only CD81 but also Pax5 resulted in an increase in the motility of HeLa cells. Thus, CD81 and Pax5-induced CD81 appeared to function as a positive regulator of cell motility (Fig. 6), which is in agreement with previous reports using dendritic, breast cancer, and B cells.

CD81 induces the facilitation of cell migration^7,8,33^. As for B cell migration and membrane protrusion, it is known that B cells migrate along the endothelium and then form actin membrane protrusions on endothelial cells, which penetrate the endothelial barrier to reach the subendothelial matrix^34^. B cell migration is composed of multiple steps, which are selectin-mediated rolling on the surface of endothelial cells, integrin-mediated adhesion to endothelial cells, and migration across the endothelial cell to the vessel wall. B cell migration and the formation of actin membrane protrusions on the B cell surface were reported to require CD81–CD19 association, which activates BCR-induced PI3K signaling^35^. We found the up-regulation of CD81 on B-lymphoma cell lines compared with neuronal and epithelial cell lines (Fig. 5). CD81 promoted the cell migration and membrane protrusion formation in HeLa cells (Fig. 6). Our data suggest that the high CD81 expression on B-lymphoma cell lines contributes to cell migration of those lymphoma cells. In addition, our results strongly support that CD81 is required for cell migration, and Pax5 is responsible for CD81 expression as a master transcription factor in B cells.

Tetraspanin CD81 was initially discovered by screening monoclonal antibodies elicited against a human B cell lymphoma for their direct anti-proliferative effects^2,36^. The monoclonal antibody, 5A6, that target CD81, inhibits the growth of B cell lymphomas in a xenograft model as effectively as rituximab, which is a standard treatment for B cell lymphoma^37^. If *CD81* gene expression could be controlled by ON or OFF Pax5-expression, Pax5-mediated CD81 regulation may be useful for the treatment of B cell lymphoma using anti-CD81 antibodies. Our study has provided insights into the molecular mechanism of *CD81* gene expression by Pax5. Our efforts to unveil the CD81 expression machinery have helped to shed new light on not only B cell development but also the improvement of molecular targeted therapy against B cell lymphoma using anti-CD81 antibodies.

## Methods

### Cell culture

Epithelial cell lines (HeLa, 293T, ME180, MCF7, A549, H1299, and SW480) and SHSY5Y human neuroblastoma cells were grown in DMEM containing 10% fetal bovine serum (FBS). B-lymphoma cell lines (Ramos, BJAB, Raji, Akata, MutuIII, and BC3) were maintained in RPMI 1640 medium supplemented with 10% FBS. Ramos, BJAB, Raji, Akata, MutuIII, SW480, and BC3 were kindly provided by Dr. S. D. Hayward (Johns Hopkins University School of Medicine, Baltimore, Maryland, USA)^38,39^. HeLa and 293T were provided by the RIKEN BioResource Research Center. ME180, MCF7, A549, and H1299 were kindly provided by Dr. H. Ariga (School of Pharmaceutical Science, Hokkaido University, Sapporo, Japan).

### Western blotting and antibodies

Cells (4 × 10^6^) were lysed by 200 μl of 4xSDS-PAGE sample buffer (containing 0.5 mM phenylmethylsulfonyl fluoride, 1 μg/ml pepstatin, and 5 μg/ml aprotinin) with/or without 1% 2-mercaptoethanol. When CD81 was detected by Western blotting with anti-CD81 antibody (Santa Cruz Biotechnology, TX, USA), the sample buffer without 2-mercaptoethanol was used. The sample was boiled for 5 min and sonicated for 30 s with a probe type sonicator in order to shear the chromosomal DNA. The resulting lysate was subjected to SDS-PAGE on 8 or 12% polyacrylamide gel followed by Western blotting^38,39^. The proteins were transferred onto a ClearTrans® nitrocellulose membrane (Wako, Osaka, Japan), and the membrane was incubated with 3% non-fat dry milk in PBS containing 0.1% Tween-20 (PBS-T) for 1 h at room temperature. Then, the membrane was incubated with a primary antibody and subsequently with a secondary antibody (horseradish peroxidase-conjugated anti-mouse or anti-rabbit IgG antibody) in Can Get Signal Immunoreaction Enhancer Solution (Toyobo, Tokyo, Japan). Primary antibodies used in these experiments included those against FLAG(DDDDK)-tag (MBL, Nagoya, Japan) and CD81, Pax5, Pax2, Pax8 and β-actin (Santa Cruz Biotechnology, TX, USA).

### Construction of luciferase reporter plasmids

Genomic DNA was isolated from BJAB cells and used as a template for amplification of the upstream promoter region of the *CD81* gene by PCR using KOD DNA polymerase (Toyobo). Amplified DNA fragments (− 1680/ + 53 CD81p) between − 1680 bp upstream and + 53 bp downstream of the transcription start site (TSS) of the *CD81* gene were cloned into the XhoI and BglII sites of the promoter-lacking firefly luciferase reporter plasmid, pGL4.11 [*luc2P*] vector (Promega, WI, USA), and the construct was designated as − 1630/ + 53 CD81p-luc. In the same way, amplified *CD81*-TSS upstream regions (− 1480/ + 53, − 1330/ + 53, − 1180/ + 53 − 1030/ + 53, − 730/ + 53, − 580/ + 53, − 430/ + 53, − 280/ + 53, − 130/ + 53, − 39/ + 53, and − 9/ + 53) were cloned into the XhoI-BglII sites of pGL4.11. The nucleotide sequences of the clones were verified by sequencing. To construct the seven-A substitutions in two potential Pax5-binding sites of − 1630/ + 53 CD81p-luc (mut-87/-81-luc, mut-54/-48-luc, and mut-87/-81&-54/-48-luc), the (-87)GCGTGAG(-81) and/or (-54)GCGGGAC(-48) of − 1630/ + 53 CD81p-luc was changed to AAAAAAA by PCR using a QuikChange Site-Directed Mutagenesis Kit (Agilent Technologies, CA, USA). The potential Pax5-binding sequence, (-87)GCGTGAG(-81), (-54)GCGGGAC(-48), and both of (-87)GCGTGAG(-81) and (-54)GCGGGAC(-48) in mut-87/-81-luc, mut-54/-48-luc, and mut-87/-81&-54/-48-luc were respectively substituted with seven (or 14) adenine nucleotides.

### Construction of CD81 and Pax5 expression plasmid

*CD81*, *PAX5,* and *PAX2* cDNAs were amplified by PCR from the cDNA library of HeLa, BJAB, and U2OS cells, respectively. *PAX8* cDNA in the pBlueScript II was provided by Dr. S. Narumi^40^. Amplified cDNAs were cloned into EcoRI and SalI sites in the pCIneo vector (Promega, WI, USA), and CD81-pCIneo and Pax5-pCIneo plasmids were constructed. To construct N-terminal 3xFLAG-tagged Pax5 (3F-Pax5-pCIneo) and C-terminal 3xFLAG-tagged Pax5 (Pax5-3F-pCIneo) plasmids, *PAX5* cDNA was obtained by EcoRI and SalI digestion from the Pax5-pCIneo and cloned into the N-terminal 3xFLAG-tag and C-terminal 3xFLAG-tag pCIneo, which were generated by the insertion of oligonucleotides encoding three repeats of FLAG-tag peptides into the front of and rear of, respectively, a multi-cloning site of pCIneo. We constructed the 2xS-tagged CD81 plasmid (2S-CD81-pCIneo) previously^15^.

### Flow cytometry

Cells were removed from culture dishes using 0.05% trypsin in 5.5 mM EDTA and washed with FACS buffer (PBS containing 3% FBS). Removed cells were fixed with 4% paraformaldehyde for 20 min. After washing with FACS buffer, cells were incubated with primary antibody in FACS buffer for 1 h at 4 °C and subsequently incubated with Alexa Fluor 647 anti-mouse secondary antibody for 1 h. Cells washed with FACS buffer were subjected to analysis by a LSRFortessa™ flow cyctometer with FACSDiva™ software (BD Biosciences, NJ, USA).

### Design of short hairpin RNA (shRNA)

Two kinds of short hairpin RNA (shRNA) targeting Pax5 (shPax5#1 and shPax5#2) were designed. The shRNA targeting of GFP (shCont) was used as a control. Sense and antisense oligonucleotides encoding shPax5#1, shPax5#2, and shGFP (shCont) were as follows: shPax5#1; 5’-GATCCCCCCGGTGATGTAGACAATAATTCAAGAGATTATTGTCTACATCACCGGTTTTTA-3’ (sense) and 5’-AGCTTAAAAACCGGTGATGTAGACAATAATTCAAGAGATTATTGTCTACATCACCGGGGG-3’ (antisense), shPax5#2; 5’-GATCCCCTAGACAATCAGTCTGTAAGTTCAAGAGACTTACAGACTGATTGTCTATTTTTA-3’ (sense) and 5’-AGCTTAAAAATAGACAATCAGTCTGTAAGTTCAAGAGACTTACAGACTGATTGTCTAGGG-3’ (antisense), and shGFP (shCont); 5’-GATCCCCTACAACAGCCACAACGTCTTTCAAGAGAAGACGTTGTGGCTGTTGTATTTTTA-3’ (sense) and 5’-AGCTTAAAAATACAACAGCCACAACGTCTTTCAAGAGAAGACGTTGTGGCTGTTGTAGGG-3’ (antisense). Sense and antisense oligonucleotides encoding shRNA were annealed and cloned into the BalII and HindIII sites of linearized pSuper^38^.

### Transfection

In the reporter assay (Figs. 1b and 2b, c), ME180 or HeLa cells (2 × 10^5^) were transfected with 1.6 μg plasmid DNA with 8 μg polyethylenimine and cultured for 24 h^41^. For B cell transfection in the reporter assay (Figs. 2d and 5b), B-lymphoma cells (4 × 10^5^) were transfected with 0.5 μg plasmid DNA using Screenfect A plus^40^ (Wako, Tokyo, JAPAN) according to the manufacturer’s instructions and cultured for 24 h. For ChIP assay, shRNA transfection, preparation of Pax5-FLAG, immunofluorescence analysis, wound healing assay, and cell migration assay, cells (1 × 10^6^) were transfected with 5 μg plasmid DNA using the Chen-Okayama calcium phosphate procedure^42^ and cultured for 24 h.

### Reporter assay

ME180 or HeLa cells (2 × 10^5^) were transfected with 1.5 μg of CD81-luciferase reporter (firefly luciferase) and 0.1 μg of pSV-β-gal (Promega) as an internal control with polyethylenimine and cultured for 24 h (Figs. 1b, 2b, and c). Harvested cells were resuspended in 0.1 ml of lysis buffer (50 mM Tris pH 7.6, 10 mM MgCl_2_, 1 mM EDTA and 0.05% NP40). Cell lysates were subjected to the firefly luciferase and β-gal assays^38,39^. Luciferase activity was measured with a GloMax 20/20 luminometer (Promega). The firefly luciferase activity was normalized to β-gal activity. For reporter assay using B cell line (Fig. 2d), BC3 cells (4 × 10^5^) were transfected with 0.2 μg CD81-luciferase reporter, 0.2 μg Pax and 0.1 μg pSV-β-gal using Screenfect A plus. Cells were cultured for 24 h and, cells resuspended in 0.1 ml lysis buffer were subjected to the firefly luciferase and β-gal assays. For dual-luciferase assay using B and epithelial cells (Fig. 5b), BJAB, ME180, and HeLa cells (4 × 10^5^) were transfected with 0.45 μg CD81-luciferase reporter (firefly luciferase) and 0.05 μg pRL-CMV-Luc (*Renilla* luciferase) as an internal control using Screenfect A plus. Cells were cultured for 24 h and, cells resuspended in 0.1 ml passive lysis buffer (Promega) for dual-luciferase assay. The activities of firefly and *Renilla* luciferases were measured with dual-luciferase reporter assay system (Promega) using a GloMax 20/20 luminometer. The firefly luciferase activity was normalized to *Renilla* luciferase activity.

### Chromatin immunoprecipitation (ChIP) assay

HeLa cells (2 × 10^6^) were transfected with 10 μg Pax5-FLAG plasmid and cultured for 12 h. Akata cells or transfected HeLa cells were treated with 1% formaldehyde for 10 min at room temperature to cross-link protein to DNA^41^. Cells were suspended in SDS-lysis buffer (1% SDS, 50 mM Tris pH 8.0, and 10 mM EDTA) with protease inhibitors (2.5 mM PMSF, 1 µM aprotinin, and 10 µM leupeptin). Soluble chromatin was sonicated with 30-s on/off pulse for 12 min and then subjected to immunoprecipitation with anti-FLAG, anti-Pax5, anti-Pax2, anti-Pax8 or mouse control antibody-immobilized beads. The DNA–Pax complex-containing immunoprecipitates were eluted using elution buffer, and the eluates were boiled at 65 °C for 4 h for dissociation of DNA–protein complex. The DNA was purified using spin columns (Fast gene, Tokyo, Japan) and subjected to PCR for amplification of the *CD81* promoter region. The sequences of PCR primer sets are shown in Table 1.

**Table 1:** DNA sequence of PCR primer for ChIP assay.

Target		DNA sequence	Fragment
− 68/ + 53 CD81p	Sense	5'-GGGCCTATGGAGGGGCGGG-3'	121 bp
	Antisense	5'-CTGGCAGGATGCGCGGTGG-3'	
− 421/− 223 CD81p	Sense	5'-AGGAAGCCCTCCCGGATTGTCCAAG-3'	199 bp
	Antisense	5'-CCAAGCTGCTGTCGCGCTCC-3'	
− 675/− 509 CD81p	Sense	5'-CTGGCCTCCTGGACACTTCACACTG-3'	167 bp
	Antisense	5'-GGGCTGGAACCTGGGCAGGCATG-3'	

### Preparation of Pax5-FLAG protein

293T cells (5 × 10^6^) were transfected with 20 μg Pax5-FLAG. After 12 h, cells were lysed in 1 ml of RIPA buffer (50 mM Tris–HCl/pH 7.4, 0.15 M NaCl, 5 mM EDTA, 1% NP-40, 0.5% sodium deoxycholate, and 0.1% SDS) containing protease inhibitors (0.5 mM NEM, 0.5 mM PMSF, 1 μg/ml aprotinin, and 1 μg/ml pepstatin). Cell lysates (1 ml) were mixed and incubated with 20 μl anti-FLAG antibody-immobilized M2 beads (Sigma-Aldrich, MI, USA) for 1 h at 4 °C. Beads were washed three times with ice-cold RIPA buffer, and Pax5-FLAG protein was eluted with 60 μl of 0.1 mg/ml 3xFLAG peptide solution. The purified Pax5-FLAG protein was used for the electrophoretic mobility shift assay.

### Oligonucleotide probes and competitors

The oligonucleotides probes, − 130/ + 53-WT, mut-87/-81, and mut-54/-48, were excised from − 130/ + 53 CD81p-luc, mut-87/-81 in − 130/ + 53 CD81p-luc, and mut-54/-48 in − 130/ + 53 CD81p-luc, respectively, by XhoI and BglII digestion and end-labeled with [α-^32^P] dCTP by Klenow DNA polymerase. ^32^P-labelled DNA probes were purified by spin columns. To prepare cold DNA probes, double-stranded oligonucleotides were generated by annealing the complementary oligonucleotides. The sequences of the upper strands of the oligonucleotides are listed in Table 2.

**Table 2:** DNA sequence of cold probe for EMSA.

Cold probe		DNA sequence	Target position
c1	Sense	5'-CCCCTCGGCTGCGCGCCCTGGCGGCAGGAGGCGGG-3'	− 130/− 99 CD81p
	Antisense	5'-CGGCCCCGCCTCCTGCCGCCAGGGCGCGCAGCCGA-3'	
c2	Sense	5'-CGGGGCCGGGGGCGGGGCGTGAGCTGGCCGGGGCG-3'	− 98/− 69 CD81p
	Antisense	5'-GCCCCGCCCCGGCCAGCTCACGCCCCGCCCCCGGC-3'	
c3	Sense	5'-GGCGGGGCCTATGGAGGGGCGGGACCGCGGCGC-3'	− 68/− 40 CD81p
	Antisense	5'-TAGGGCGCCGCGGTCCCGCCCCTCCATAGGCCC-3'	

### Electrophoretic mobility shift assay (EMSA)

Purified Pax5-FLAG protein was incubated in binding buffer (10 mM Tris–HCl [pH 7.5], 50 mM NaCl, 0.5 mM EDTA, 4% glycerol, 40 ng of poly(dI-dC), and 0.5 mM DTT) for 10 min at 25 °C. These samples were reacted with the ^32^P-labelled DNA probe for 20 min at 25 °C. The reaction mixture was run on 6% non-denaturing polyacrylamide gel in 0.5xTBE buffer containing tris borate buffer (pH 8.5) in 1.25 mM EDTA at 300 V. The gel was dried and exposed overnight to X-ray film. For competition EMSA, a 100-fold molar excess of the cold probes was added to the EMSA binding reaction.

### Immunofluorescence analysis (IFA)

Cells on a glass slide were fixed with 4% paraformaldehyde at room temperature for 10 min and permeabilized with 0.1% Triton X-100 in PBS. The samples were treated with 3% bovine serum albumin in PBS and incubated with Alexa488-conjugated phalloidin (F-actin) and mouse anti-S-probe or mouse anti-FLAG monoclonal antibodies. After washing, the cells were further incubated with Alexa fluor 594 conjugated anti-mouse IgG. Nuclei were visualized using DAPI. Stains were evaluated with a confocal LSM 800 microscope (Carl Zeiss, Oberkochen, Germany) using the LSM software.

### Wound healing assay

Transfected HeLa cells were plated in 6-well plates, wounded by scratching with a pipette tip, then incubated with DMEM containing 10% FBS for 24 h. Cells were photographed using a phase-contrast microscope (Olympus, Tokyo, Japan). The wound area was calculated by ImageJ software^43^ (NIH, MD, USA). Wound area filled = (mean of first wound area – mean of last wound area)/first wound area × 100.

### Cell migration assay

HeLa cells transfected with CD81 or Pax5 were cultured for 24 h. Harvested cells (10^5^ cells) were seeded on an upper well of Transwell (4.0 µm pore size) and cultured in a serum-free DMEM. Then, DMEM containing 10% FBS was added to the bottom well plate. After 48 h, the cells in the upper surface of the membrane were removed with a cotton swab. The migrated cells in the lower chamber were fixed, and genome of migrated cells were stained by DAPI. The number of these cells was counted manually.

### Proliferation assays

Cell proliferation was measured by using a Cell Count Reagent SF (Nacalai Tesque Inc. Kyoto, Japan). Briefly, cells were plated in 96-well plates at an initial density of 2.5 × 10^4^ cells/well in 100 μl of medium. To measure cell proliferation at 0, 24, and 48 h after seeding at 37 °C, 10 µl of the Cell Count Reagent SF solution was added in each well. The cells were incubated at 37 °C, and 1 h later, the optical density at 450 nm of each sample was measured using a microplate spectrophotometer (Tecan M200; Tecan, Kanagawa, Japan). Data are shown as the means ± SEM of three independent experiments.

### Densitometry and statistical analyses

Densitometric analysis of the blotting data was performed using ImageJ software. The statistical significance between each group and the control (empty vector-transfected cells) was analyzed by one-way ANOVA followed by Dunnett's or Tukey’s test for multiple comparisons. Statistical significance was tested using GraphPad Prism 7 (GraphPad Software, CA, USA). ***P < 0.001 and ****P < 0.0001 indicate statistical significance, and “n.s.” indicates not significant.

## Supplementary Information

Supplementary Information.

## Data Availability

To identify the promoter region of the *CD81* gene, the upstream DNA sequence (1.63 kbp) of the NCBI Reference Sequence (NG_023386.1) for the *CD81* gene was taken from NCBI-nucleotide (ULT: https://www.ncbi.nlm.nih.gov/nucleotide/). The transcriptional start site (TSS) of the *CD81* gene was defined by reference to the DataBase of Transcriptional Start Sites (DBTSS) software ver10.1 (URL: https://dbtss.hgc.jp/). The start codon (+ 234) in the first exon was shown by the NCBI Reference Sequence (NM_004356.4). The binding sites of transcription factors in the upstream DNA sequence of the *CD81* gene were analyzed by the open-source software, JASPER database (7th release) (URL: http://jaspar.genereg.net/). The sequence logo of Pax5, Pax2 and Pax8 consensus binding sequences were generated using ISMARA URL: (https://ismara.unibas.ch/mara/). Original images of ChIP assay and Western blotting are shown in Supplementary Fig. S2–S5. Other data are available from the corresponding author upon reasonable request.
