# Supplementary material for: Pax5 mediates the transcriptional activation of the CD81 gene
Source: Sci Rep. 2021 Nov 25;11:22919. doi: 10.1038/s41598-021-02082-9 (PMC8616915; doi:10.1038/s41598-021-02082-9)

Supplementary information

Pax5 mediates the transcriptional activation of the *CD81* gene

Kohei Hosokawa, Hanako Ishimaru, Tadashi Watanabe and Masahiro Fujimuro*

Department of Cell Biology, Kyoto Pharmaceutical University,
Misasagi-Shichono-cho 1, Yamashina-ku, Kyoto 607-8412, Japan.

*: Address correspondence to Masahiro Fujimuro, PhD.
Department of Cell Biology, Kyoto Pharmaceutical University
Misasagi-Shichono-cho 1, Yamashina-ku, Kyoto 607-8412, Japan;
Tel: +81-75-595-4717
E-mail: fuji2@mb.kyoto-phu.ac.jp

Supplementary Figure S1

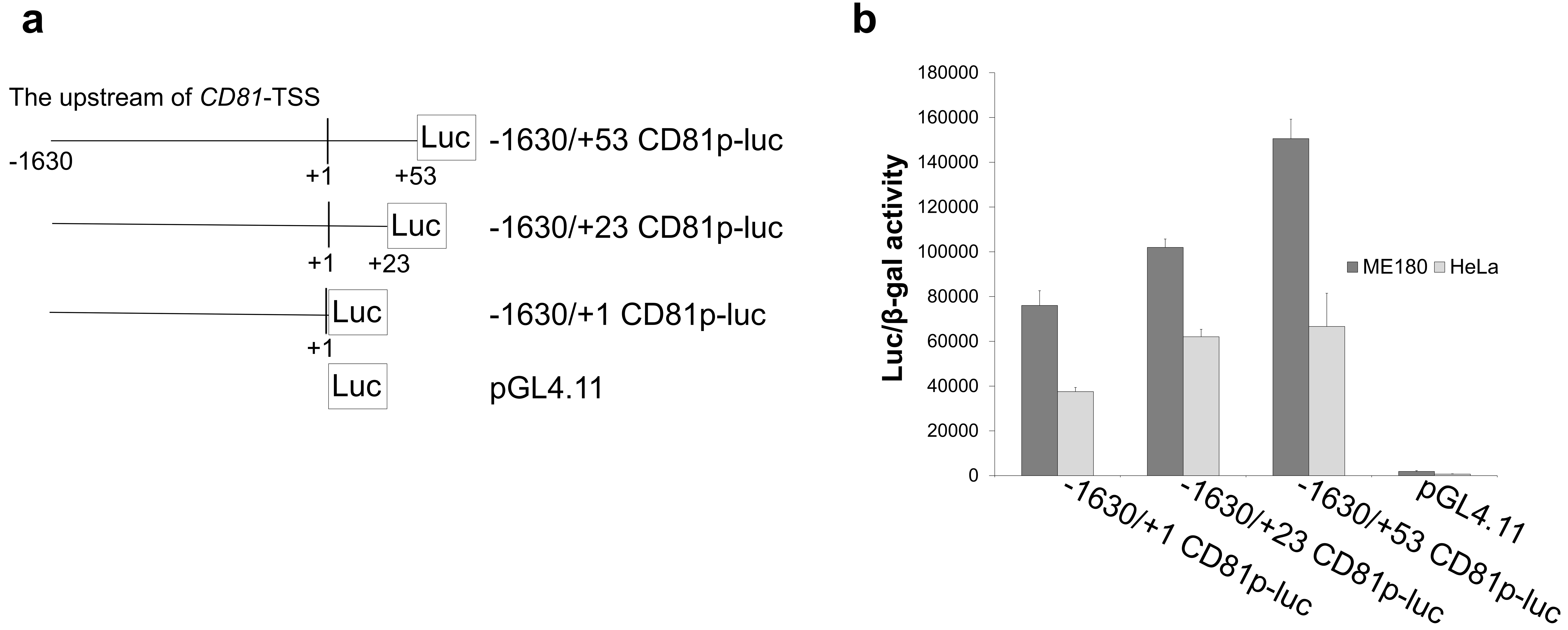

Supplementary Figure S1. Identification of the *CD81* promoter region

(A) The schematic diagrams of luciferase reporter plasmids to evaluate the transcriptional activity of the *CD81* gene 3'-flanking region. The transcriptional start site (TSS) of the *CD81* gene is defined as +1, and the luciferase gene is indicated as "*Luc*". DNA fragments from *CD81*-gene upstream -1630 bp to *CD81*-TSS region +53 bp, +23 bp, and +1 bp were cloned into the promoter-lacking luciferase reporter plasmid (pGL4.11 [luc2P]), and constructs were designated as -1630/+53CD81p-luc, -1630/+23CD81p-luc, and -1630/+1CD81p-luc, respectively. (B) Transcriptional activity of the *CD81* gene 3'-flanking region. ME180 and HeLa cells were transiently transfected with the reporter and pSV-β-gal plasmids, and the luciferase activity was measured at 48 h after transfection. Transfection efficiency was normalized by the β-gal activity. Luciferase/β-gal activity means the relative light units of Luc divided by β-gal.

Supplemental Figure S2 (original images of Figure 3a, 3b and 3d)

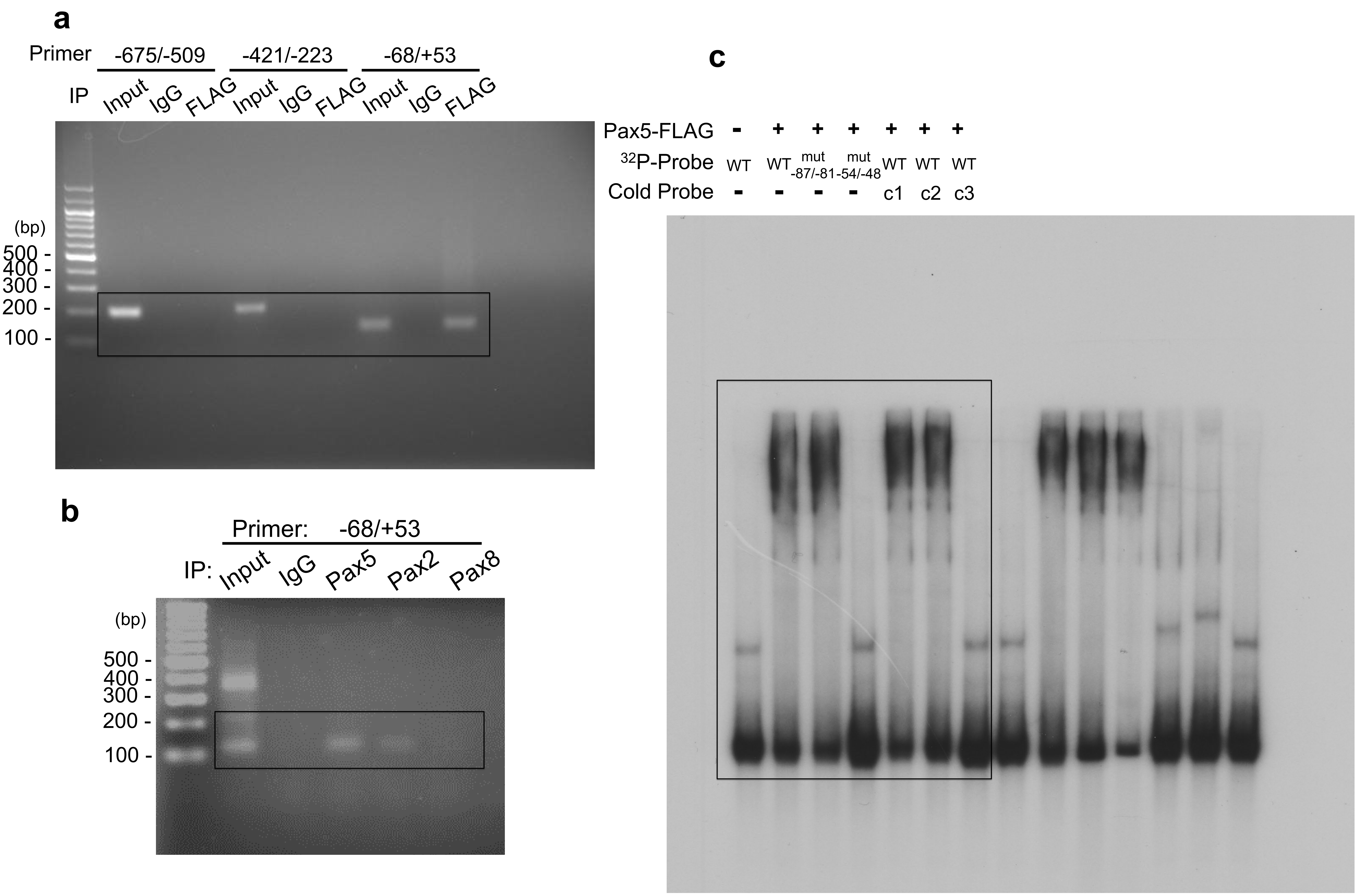

Supplemental Figure S3 (original images of Figure 4b and 4c)

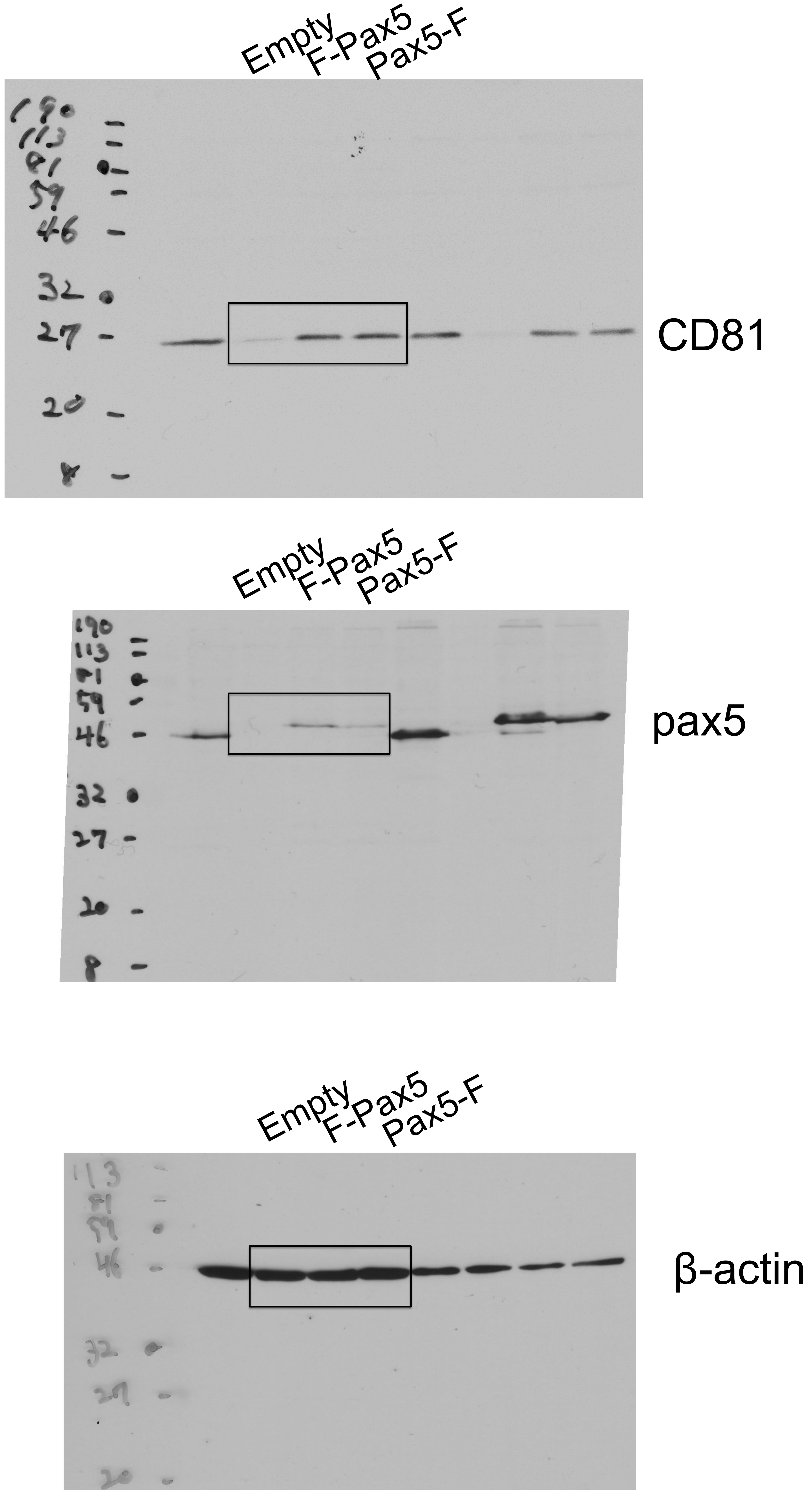

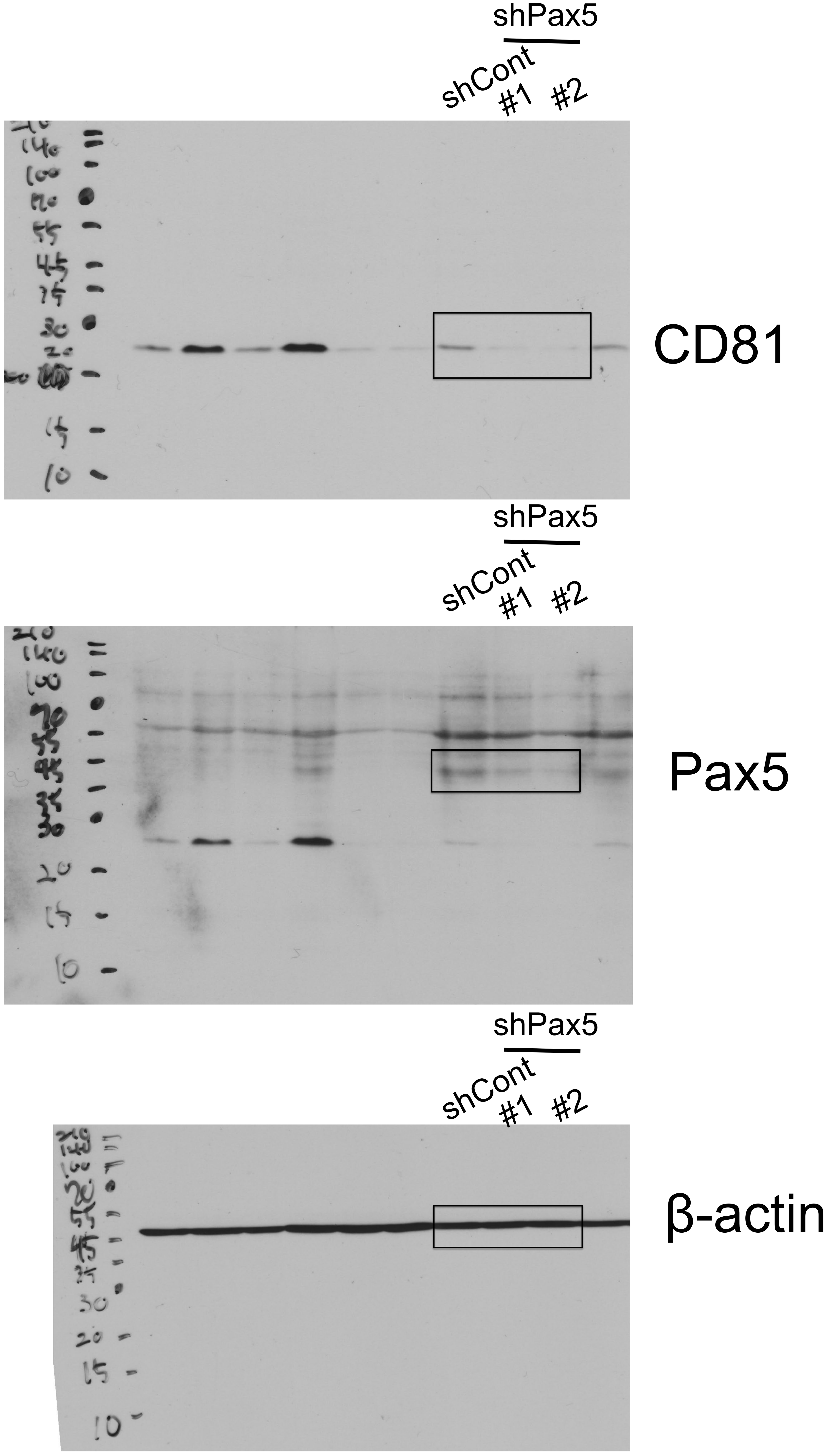

Supplemental Figure S4, 1/2 (original images of Figure 5a)

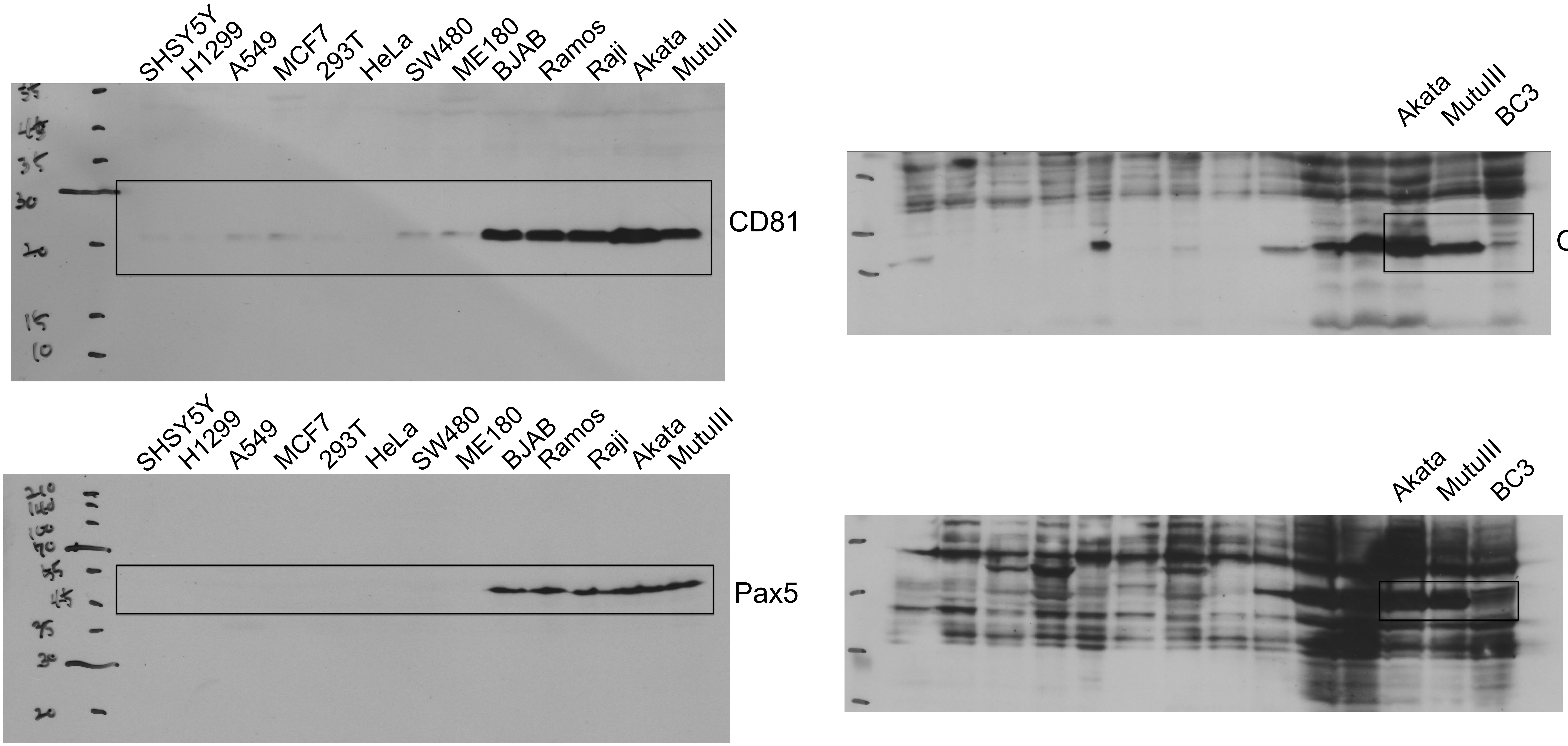

Supplemental Figure S4, 2/2 (original images of Figure 5a)

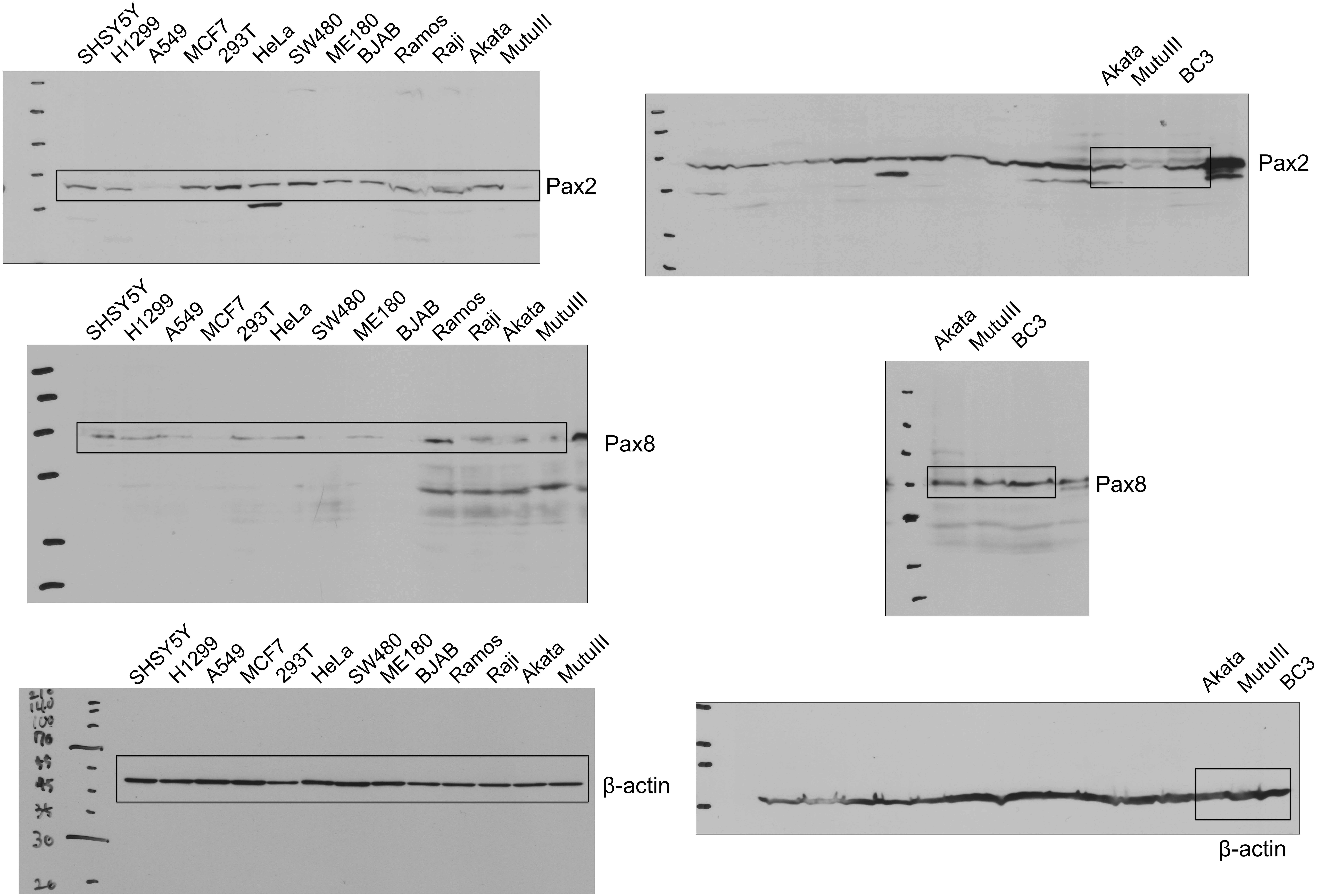

Supplemental Figure S5

The long exposure images of CD81 or Pax5 blotting using ME180, H1299, and HeLa extracts

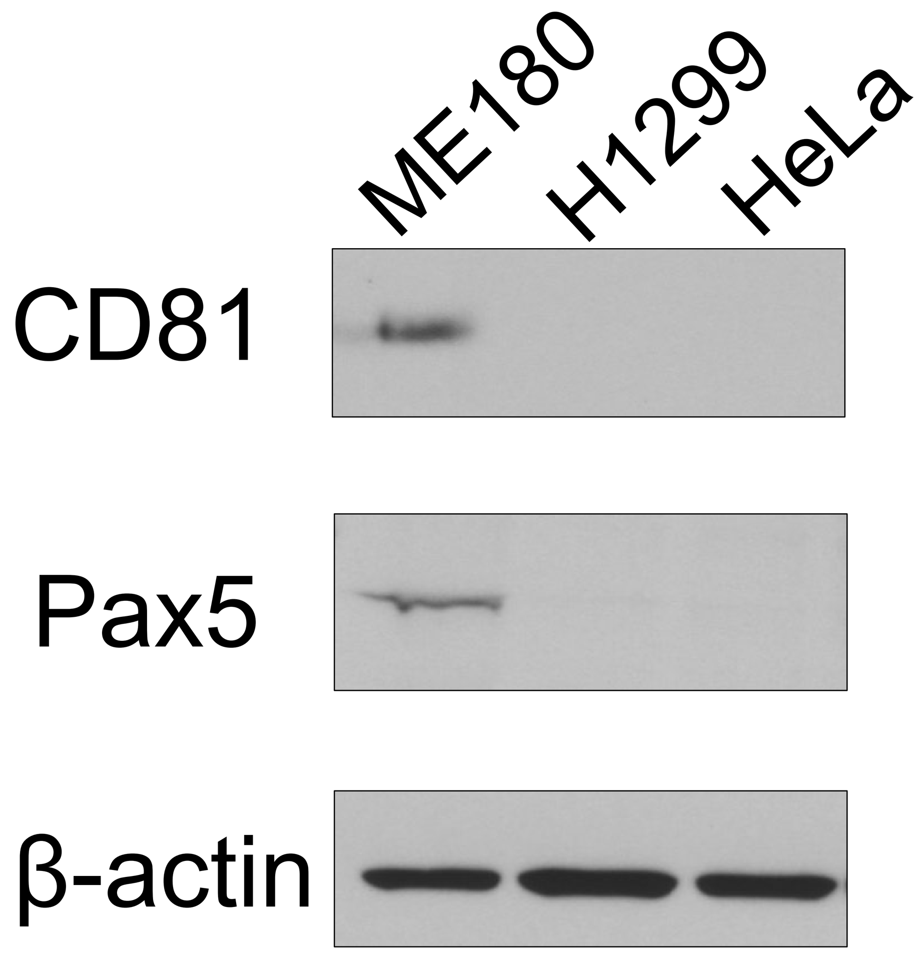

(original images of above blotting)

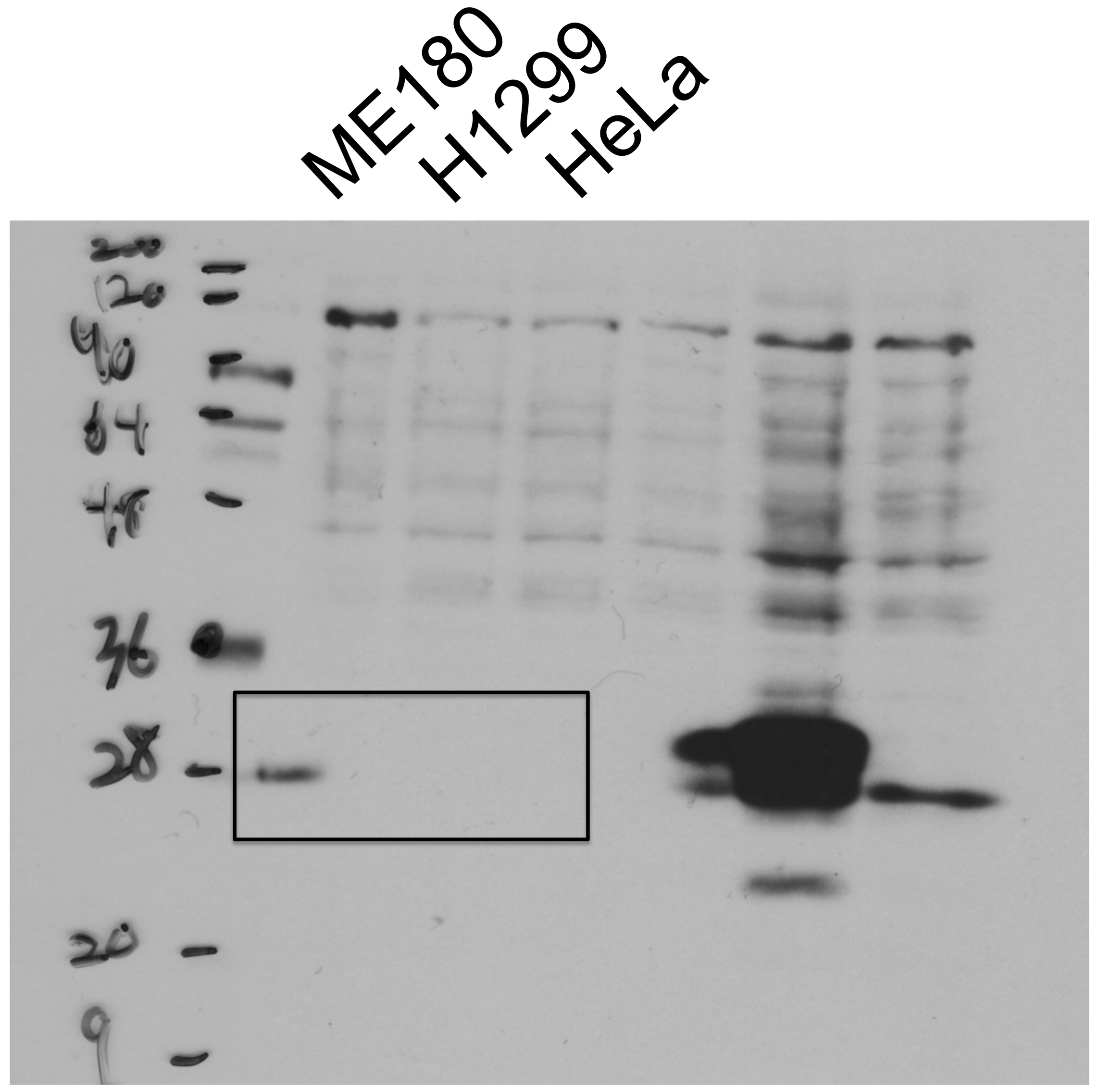

CD81(Long exposure)

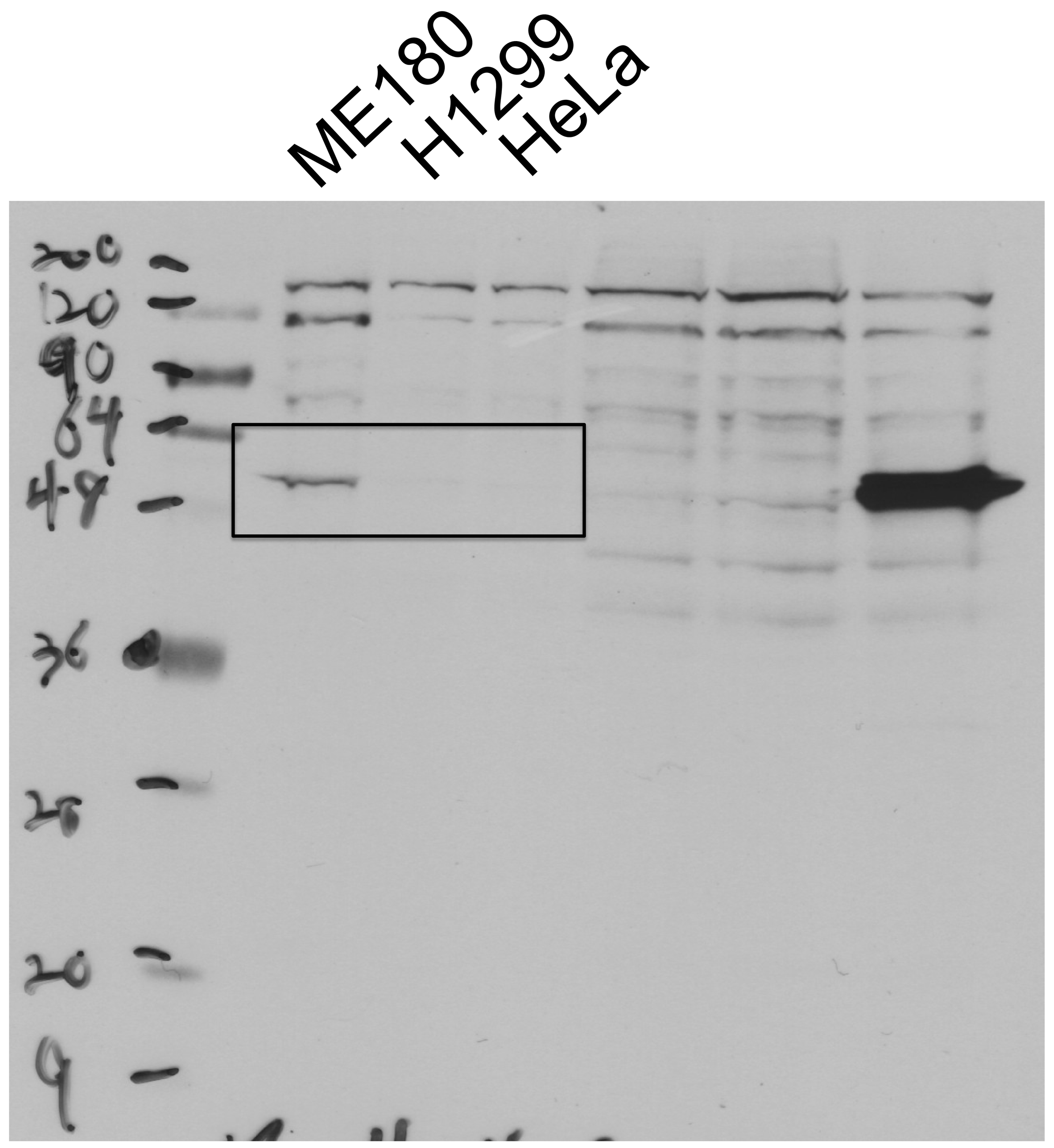

Pax5 (Long exposure)

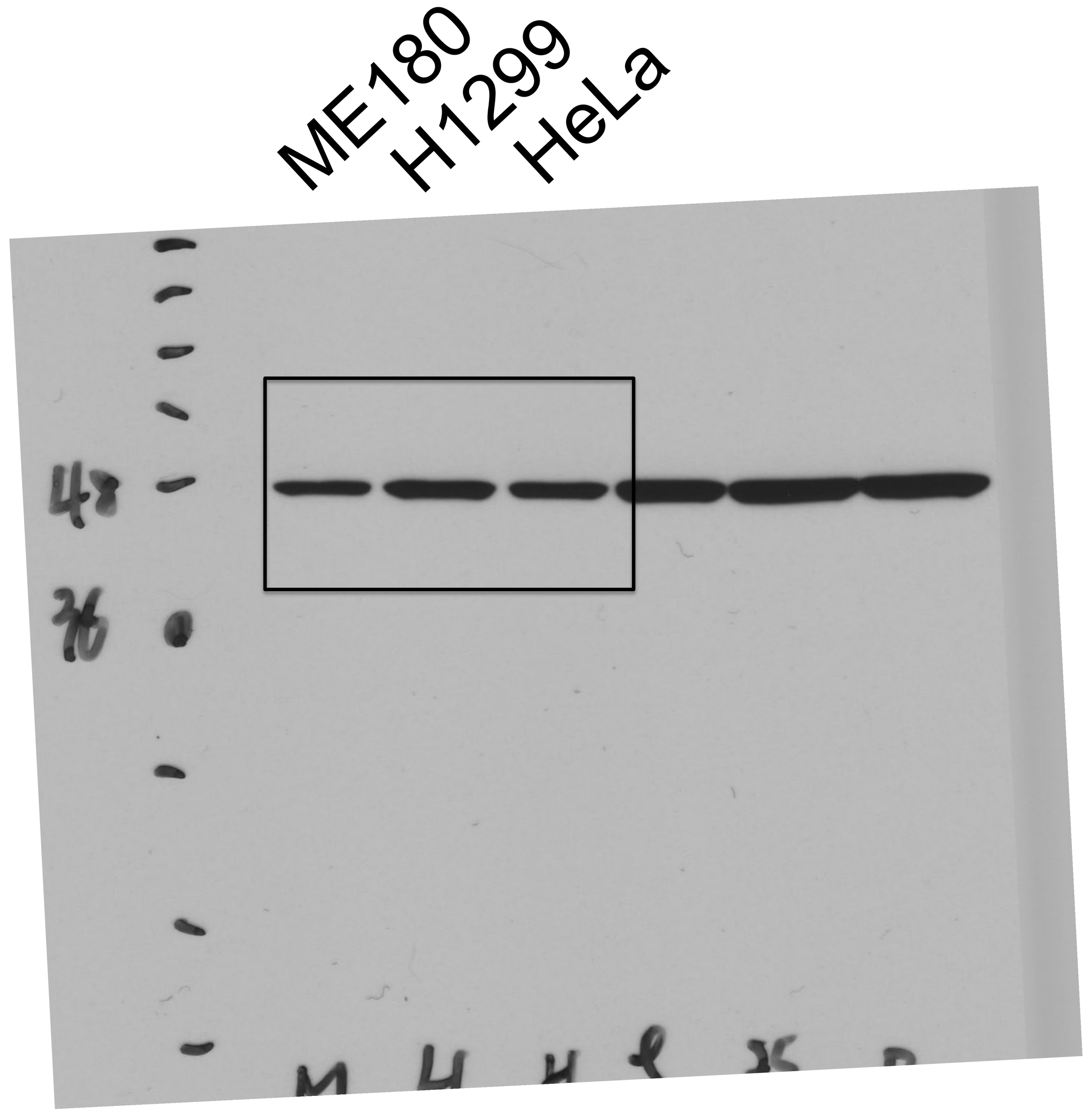

β-actin

Supplemental Figure S6 (original images of Figure 6e and 6f)

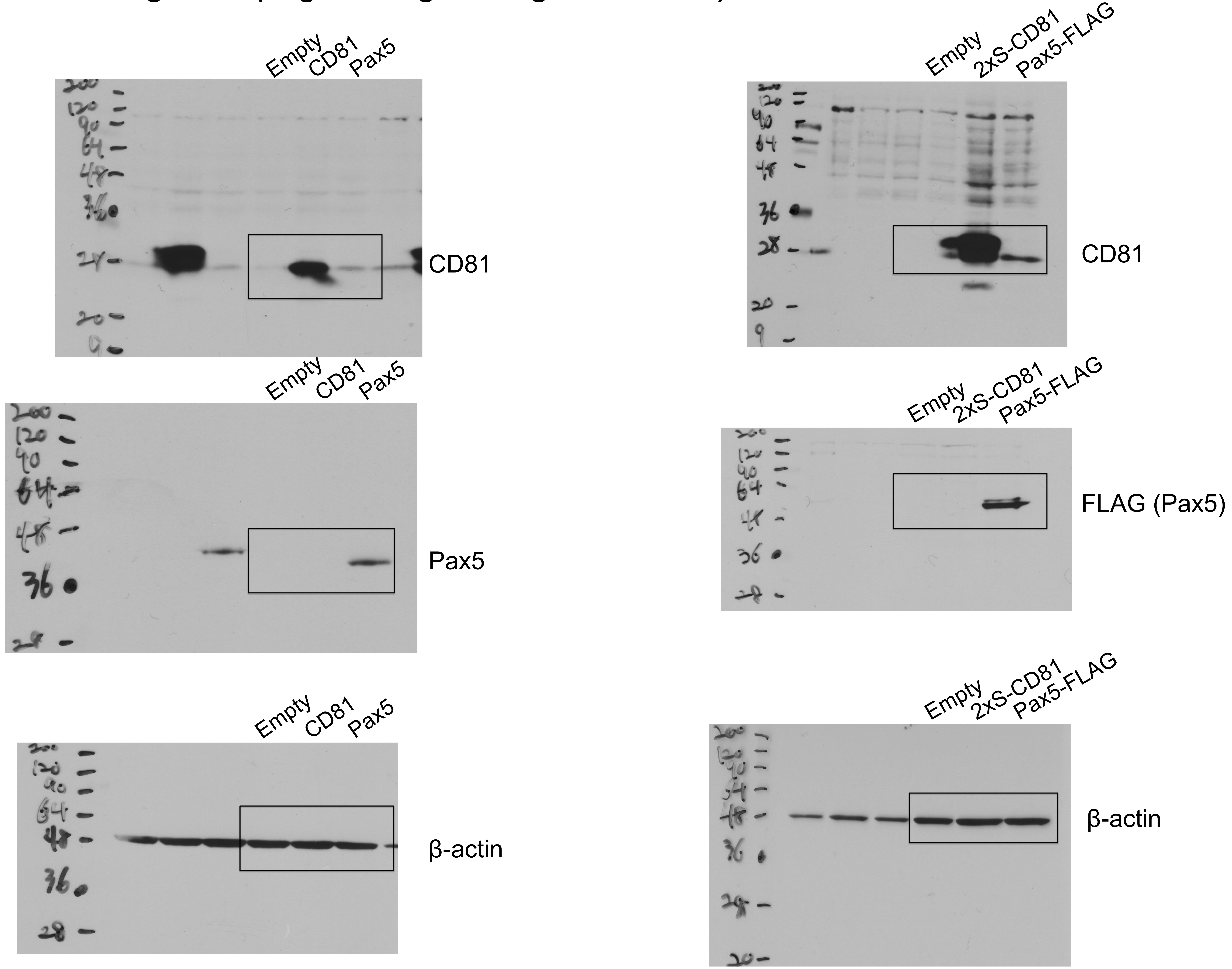

Supplement: Supplementary file 1 — Supplementary Information. [file 41598_2021_2082_MOESM1_ESM.pdf]
